# Integrated Chemical Profiling, Serum Pharmacochemistry, and Network Pharmacology to Elucidate the Anti-Hyperlipidemic Effects of *Rosa laxa* Retz. Fruits from Different Geographical Origins

**DOI:** 10.3390/metabo16060392

**Published:** 2026-06-04

**Authors:** Yuan He, Qianqian Feng, Wenhui Zhao, Li Tian

**Affiliations:** 1 College of Pharmacy, Xinjiang Medical University, Urumqi 830017, China; mr.hymail@gmail.com; 2 College of Traditional Chinese Medicine, Xinjiang Medical University, Urumqi 830017, China; fqq0710@163.com; 3 Science & Technology Innovation and Transformation Service Center, Xinjiang Medical University, Urumqi 830017, China; 4 Institute of Traditional Chinese Medicine, Xinjiang Medical University, Urumqi 830017, China

**Keywords:** fruits of *Rosa laxa* Retz., hyperlipidemia, compound identification, pharmacodynamics, mechanism of action

## Abstract

Background: The fruits of *Rosa laxa* Retz. (FRL) is a traditional medicinal and edible fruit widely used in Xinjiang for its potential health benefits. Its chemical variations across geographical origins remain poorly understood, as do the molecular mechanisms underlying its anti-hyperlipidemic effects. This study aimed to characterize the chemical profile of FRL extract (FRLE) from different origins, identify its bioactive constituents and metabolites in vivo, and evaluate its efficacy and potential mechanisms against HLP. Methods: UPLC-QTOF-MS was employed for qualitative and quantitative profiling, combined with PCA to differentiate samples from five origins. An HLP mouse model was established to evaluate the pharmacodynamic effects, while acute and sub-chronic toxicity tests assessed safety. Serum pharmacochemistry was used to track absorbed constituents and metabolites. Finally, network pharmacology, molecular docking, and Western blot were integrated to elucidate the underlying mechanisms. Results: A total of 60 compounds were identified in FRLE, with 20 key components quantified via the TOF-MRM mode. PCA indicated that the Yamalike Mountain samples possessed the most diverse chemical profile and the highest response of active markers. Pharmacodynamic results showed that FRLE (extraction yield 24.19%) significantly improved TC, LDL-C, and corrected abnormal HDL-C levels in HLP mice, while H&E staining confirmed the alleviation of hepatic steatosis. Safety evaluations revealed no significant acute or cumulative toxicity at the maximum feasible dose of 16.6 g/kg. In rat plasma, 15 prototypes and 14 metabolites were identified. FRLE acted on the “Lipid and Atherosclerosis” pathway by modulating key targets, including NFE2L2, CYP1A1, NOS3, and MAPK1. Conclusions: Our findings demonstrate that FRLE is a safe and effective candidate for the management of hyperlipidemia. This study establishes a link between the material basis and biological mechanisms of FRL, thereby providing a scientific foundation for its further resource development and clinical application.

## 1. Introduction

Hyperlipidemia (HLP) is a chronic metabolic disorder characterized by abnormal serum lipid profiles [1]. According to the latest data from the World Health Organization, approximately 39% of adults worldwide exhibit dysregulated total cholesterol levels. This condition is a primary driver of atherosclerotic cardiovascular disease, which causes nearly 17.9 million deaths annually and accounts for 32% of all global mortality [2]. Statins and fibrates remain the cornerstone of HLP pharmacotherapy, yet their long-term clinical utility is often constrained by adverse effects [3]. Chronic statin administration is associated with muscle-related complications, ranging from myalgia and myositis to life-threatening rhabdomyolysis [4,5]. Similarly, prolonged fibrate therapy has been linked to an increased incidence of cholelithiasis and gastrointestinal disturbances [6,7,8]. These safety concerns underscore the need to develop novel lipid-lowering agents that are both effective and suitable for long-term administration.

*Rosa laxa* Retz., a distinctive botanical resource native to Xinjiang, is widely distributed and exhibits considerable stress resistance. The mature fruit of *Rosa laxa* Retz. (FRL), known as Fructus Rosae Laxae, is formally recorded in the Pharmacopoeia of Kazakh Medicine [9]. It is traditionally utilized in the preparation of various functional foods, such as jams, syrups, and beverages [10]. Previous studies have demonstrated that FRL extracts exhibit significant hepatoprotective activity in murine models of acute toxic hepatitis [9]. Our preliminary research indicated that FRL is rich in bioactive constituents, such as flavonoids, polyphenols, organic acids, polysaccharides, and amino acids, which possess potent lipid-regulating properties [11]. The rapid evolution of pharmacological and disease-related databases has drawn increasing attention to bioinformatics. In recent years, network pharmacology has emerged as a powerful tool in the study of Traditional Chinese Medicine (TCM) and natural products [12]. This approach facilitates the construction of complex interaction networks encompassing “component-target-disease-pathway”, thereby enabling a systematic exploration of the pharmacological mechanisms underlying multi-component therapies [13,14]. Network pharmacology thus provides a robust framework to further elucidate the molecular mechanisms by which the chemical constituents in FRL extract (FRLE) exert their lipid-lowering effects.

This study aimed to systematically evaluate the therapeutic potential and regulatory mechanisms of FRLE against hyperlipidemia. Samples from Yamalike Mountain were specifically selected for detailed chemical composition analysis because preliminary principal component analysis (PCA) and statistical evaluations revealed that FRL from this region possessed the greatest abundance of high-response chemical constituents. To achieve this central aim without overextending our objectives, the research was executed through a three-stage sequential workflow. First, the chemical profile of FRLE was comprehensively characterized using liquid chromatography-mass spectrometry (LC-MS) to map its primary active constituents. Second, a hyperlipidemia mouse model was established to evaluate the in vivo lipid-lowering efficacy of the extract derived from this optimal origin, while its safety profile was rigorously investigated through acute and sub-chronic toxicity assays in healthy mice. Finally, the underlying molecular mechanisms of these identified constituents were predicted via an integrated, hypothesis-generating approach of network pharmacology and molecular docking, followed by empirical validation using Western blotting. Collectively, this systematic pipeline bridges the gap between raw material profiling, phenotypic efficacy, and molecular execution, providing a novel and structured strategy for discovering effective preventive and therapeutic agents for hyperlipidemia derived from TCM.

## 2. Materials and Methods

### 2.1. Chemicals and Reagents

Optima-grade methanol was purchased from Thermo Fisher Scientific (Waltham, MA, USA), and distilled water was obtained from Watson’s (Hong Kong, China). Reference standards, including quercetin, euscaphic acid, protocatechuic aldehyde, citric acid, quinic acid, hyperoside, astragalin, kaempferol, kaempferol-3-O-rutinoside, oleanolic acid, asiatic acid, ursolic acid, corilagin, and epicatechin, were purchased from Chengdu Must Bio-Technology Co., Ltd. (Chengdu, China). Malic acid was obtained from Shanghai Standard Technology Service Co., Ltd. (Shanghai, China). Pomolic acid and tartaric acid were purchased from Baoji Herbest Bio-Tech Co., Ltd. (Baoji, China). Raffinose, arginine, and sucrose were sourced from Shanghai Chunyou Bio-Technology Co., Ltd. (Shanghai, China). Rutin, tiliroside, tormentic acid, and isoquercitrin were purchased from Chengdu Herbpurify Co., Ltd. (Chengdu, China). Ellagic acid and gallic acid were obtained from Sichuan Vicky Biotechnology Co., Ltd. (Chengdu, China), while luteoloside was provided by the National Institutes for Food and Drug Control (Beijing, China). The purity of all reference standards was verified to be >98%. Absolute ethanol was purchased from Tianjin Xinbote Chemical Co., Ltd. (Tianjin, China). Sodium formate was obtained from Merck (Darmstadt, Hesse, Germany), and leucine-enkephalin was purchased from Waters Corp. (Milford, MA, USA).

Regarding biological reagents, the 40% kcal high-fat diet (HFD) was purchased from Beijing Boaigang Biological technology (Beijing, China). Commercial kits for total cholesterol (TC), triglycerides (TG), high-density lipoprotein cholesterol (HDL-C), and low-density lipoprotein cholesterol (LDL-C) were obtained from Nanjing Jiancheng Bioengineering Institute (Nanjing, China). Rosuvastatin calcium tablets were purchased from Simcere Pharmaceutical Co., Ltd. (Nanjing, China), and fenofibrate capsules were from Abbott Laboratories (Shanghai, China). Primary antibodies against NFE2L2 (Cat No. 80593-1-RR), NOS3 (Cat No. 27120-1-AP), CYP1A1 (Cat No. 13241-1-AP), PPARG (Cat No. 16643-1-AP), and MAPK1 (Cat No. 51068-1-AP) were purchased from Proteintech Group (Wuhan, China). The GAPDH (Cat No. GB11002-100) antibody was obtained from Servicebio Technology Co., Ltd. (Wuhan, China). Additional supplies, including the one-step gel casting kit, were purchased from Shanghai Yaemei Biotechnology Co., Ltd. (Shanghai, China); the tri-color pre-stained protein marker was from Life-ilab Co., Ltd. (Shanghai, China); the ECL chemiluminescence kit was from Beijing Lanjieco Technology Co., Ltd. (Beijing, China); and PVDF membranes were purchased from Roche (Basel, Switzerland).

### 2.2. Preparation of FRLE Samples

FRLs were collected from five distinct locations: Yamalike Mountain in Urumqi, the Urumqi Botanical Garden, Hotan Prefecture, Altay Region, and Habahe County. The maturity stage at harvest was determined based on morphological criteria, specifically targeting fruits with a uniform deep red color, and all samples were collected annually in mid-September. All specimens were authenticated as the mature fruits of *Rosa laxa* Retz. by Professor Haiyan Xu of Xinjiang Medical University. Voucher specimens were deposited in the Laboratory of Extraction and Separation of Traditional Chinese Medicine Constituents at Xinjiang Medical University. The sepals and pedicels were removed, and the fruits were halved to eliminate seeds and trichomes. The processed fruits were air-dried, pulverized, and passed through a 50-mesh sieve to obtain FRL powder for subsequent use.

The FRL powder was extracted using a reflux method. Briefly, a specific amount of the dry powder was macerated overnight in 65% ethanol at a solid-to-liquid ratio of 1:18 (*w*/*v*), followed by reflux extraction for 1.5 h. After filtration, the residue was re-extracted with 65% ethanol at a ratio of 1:17 (*w*/*v*) for 1 h. The filtrates from both extractions were combined, concentrated under reduced pressure, and vacuum-dried at 70 °C to yield a brownish-black powder, designated as FRLE. The extraction yield was 24.19% (*w*/*w*).

To prepare the analytical samples, approximately 5.0 mg of FRLE powder was accurately weighed and completely dissolved in 65% chromatographic-grade methanol. The solution was transferred to a 10 mL volumetric flask and diluted to volume. The mixture was then subjected to high-speed centrifugation at 13,780 *g* for 10 min. The resulting supernatant was collected as the FRLE sample for further analysis.

### 2.3. UPLC-QTOF-MS Analysis of Chemical Constituents in FRLE from Different Origins

The FRLE samples were separated using an ultra-performance liquid chromatography (UPLC) system (I-Class, Waters, Milford, MA, USA) coupled with a quadrupole time-of-flight mass spectrometer (QTOF-MS; Xevo G2-XS, Waters, Milford, MA, USA) equipped with an electrospray ionization (ESI) source.

Chromatographic conditions: Samples were injected into an ACQUITY UPLC HSS T3 column (2.1 mm × 100 mm, 1.8 µm; Waters) for separation. The column temperature was maintained at 40 °C, and the injection volume was 1 µL. The flow rate was set at 0.2 mL/min. The mobile phase consisted of methanol (A) and 0.1% formic acid in water (*v*/*v*, B). The gradient elution program was optimized as follows: 0–3 min, 0% A; 3–20 min, 0–45% A; 20–23 min, 45% A; 23–28 min, 45–100% A; 28–31 min, 100% A; 31–36 min, 100–0% A; and 36–39 min, 0% A.

Mass spectrometry was performed in MS^E^ mode. For ESI (+), the parameters were set as follows: source temperature, 120 °C; capillary voltage, 3.5 kV; sampling cone voltage, 40 V; source offset voltage, 80 V; cone gas flow, 50 L/h desolvation temperature, 450 °C; and desolvation gas flow, 900 L/h. The collision energy was set at 6 eV for the low-energy function and ramped from 20 to 60 eV for the high-energy function. For real-time mass calibration, leucine-enkephalin was utilized as the lock-spray (ESI+: *m*/*z* 556.2771; ESI-: *m*/*z* 554.2615), and 5 mM sodium formate was used for multi-point external calibration. The ESI (−) parameters were identical to those of ESI (+), except that the capillary voltage was adjusted to 2.5 kV.

Data acquisition and processing, including the analysis of constituent differences across various origins, were conducted using Progenesis QI software (Ver. 3.0, Waters, Milford, MA, USA).

### 2.4. Chemical Constituent Analysis of FRLE

The FRLE samples exhibiting the highest number of chemical constituents among the five different origins was selected for further investigation. The corresponding MS^E^ raw data were imported into the UNIFI informatics platform (Ver. 1.9.4, Waters, Milford, MA, USA). Chemical constituents within FRLE were identified by matching against an in-house chemical information database.

The in-house database was established as follows: comprehensive literature reviews were conducted to collect mass spectrometry data (including precursor and fragment ion information) of other medicinal plants within the same genus (*Rosa*). The structural information of these compounds in .mol format was retrieved from the ChemSpider database (https://www.chemspider.com/, accessed on 31 May 2026). These structural files were subsequently imported into the UNIFI compound library, where the collected precursor and fragment ion data were appended as supplementary mass spectrometry parameters.

Compound identifications tentative by UNIFI were further validated by comparing their retention times and MS/MS fragmentation patterns with reference standards using the UPLC-QTOF-MS/MS method. Finally, the confirmed positive constituents were quantitatively analyzed using the TOF-MRM (Multiple Reaction Monitoring) mode.

To establish the calibration curves, reference standards were precisely weighed and dissolved in methanol. Stock solutions of astragalin and oleanolic acid were prepared at 0.5 mg/mL, while ellagic acid, tartaric acid, citric acid, gallic acid, pomolic acid, quercetin, kaempferol, kaempferol-3-O-rutinoside, arginine, rutin, hyperoside, euscaphic acid, malic acid, isoquercitrin, quinic acid, epicatechin, tiliroside, and protocatechuic aldehyde were prepared at 0.1 mg/mL. These stock solutions were serially diluted to generate a range of working concentrations: astragalin and oleanolic acid (500, 250, 125, 62.5, and 30.125 μg/mL); citric acid and pomolic acid (100, 50, 25, 12.5, and 6.25 μg/mL); ellagic acid and arginine (50, 25, 12.5, 6.25, and 3.125 μg/mL); tartaric acid, gallic acid, quercetin, epicatechin, euscaphic acid, isoquercitrin, quinic acid, and malic acid (10, 2.5, 0.625, 0.3125, and 0.15625 μg/mL); kaempferol, kaempferol-3-O-rutinoside, hyperoside, and tiliroside (1000, 500, 250, 125, and 62.5 ng/mL); and protocatechuic aldehyde and rutin (100, 50, 25, 12.5, and 6.25 ng/mL).

### 2.5. Pharmacodynamic Evaluation of FRLE Against HLP

Institute for Cancer Research (ICR) mice (6 weeks old, weighing 20 ± 2 g) were obtained from the Animal Experiment Center of Xinjiang Medical University [License No. SCXK (Xin) 2023-001]. The animals were housed in a Specific Pathogen-Free (SPF) facility [License No. SYXK (Xin) 2023-004] under controlled conditions: temperature at 24 °C, relative humidity at 50%, and a 12 h light/dark cycle, with ad libitum access to food and water. All experimental procedures were conducted in accordance with the Guide for the Care and Use of Laboratory Animals and approved by the Animal Ethics Committee of the First Affiliated Hospital of Xinjiang Medical University (Approval Nos. IACUC-20240308-21).

After one week of acclimatization, the mice were randomly assigned to the control group (*n* = 6) and the HLP model group (*n* = 54). The control group was fed a standard diet, while the model group received an HFD for 8 weeks. Subsequently, blood was collected from the retro-orbital venous plexus, and serum was isolated by centrifugation at 3000 rpm for 15 min. Success of the HLP model was confirmed by comparing the four primary lipid profiles (TC, TG, HDL-C, and LDL-C) between the two groups.

Successfully modeled mice were randomly subdivided into 8 groups (*n* = 6 per group): model, fenofibrate (Fb, 26 mg/kg), rosuvastatin (Rt, 1.3 mg/kg), Yamalike Mountain FRLE low-dose (FRLE-L, 1.66 g/kg), Yamalike Mountain FRLE medium-dose (FRLE-M, 8.30 g/kg), Yamalike Mountain FRLE high-dose (FRLE-H, 16.60 g/kg), Hotan FRLE (FRLE-HT, 16.60 g/kg), and Altay FRLE (FRLE-ALT, 16.60 g/kg). The control group and model group received sterile saline via oral gavage. Dosages for Fb, Rt, and FRLE-L were calculated based on human equivalent doses according to body surface area, as referenced in clinical guidelines and the Pharmacopoeia of Kazakh Medicine. Interventions lasted for 5 weeks, with body weights recorded weekly. Upon study completion, mice were anesthetized, and blood was collected via retro-orbital bleeding. Serum lipid levels were measured using commercial kits. Livers were excised, rinsed with saline, blotted dry, and weighed to calculate the liver index as follows: Liver Index = (liver weight/body weight) × 100% [15].

For histopathological examination, the large lobes of the livers were fixed in a universal tissue fixative for 24 h. Tissues were then trimmed (10 mm × 20 mm), dehydrated through a graded ethanol series, cleared in xylene, and embedded in paraffin. Sections (4 μm) were cut, mounted, and dried. Following deparaffinization and rehydration, sections were stained with Hematoxylin and Eosin (H&E), cleared, and mounted with neutral balsam [16]. Images were captured using an automated slide scanner, utilizing 10× magnification for coarse focusing (minimum 3 focus points per slide) and 20× magnification for fine focusing (minimum 5 focus points per slide).

### 2.6. Safety Evaluation of FRLE

ICR mice (6 weeks old, weighing 20 ± 2 g) were housed in an SPF facility under controlled conditions: 24 °C, 50% relative humidity, and a 12 h light/dark cycle, with ad libitum access to food and water. As FRL is recognized as a medicinal and edible resource, an acute toxicity study was conducted using the maximum feasible dose. Based on the maximum solubility of FRLE in water (830 mg/mL), a dose of 16.6 g/kg body weight was administered via oral gavage three times at 4 h intervals. Following administration, the clinical signs, behavioral patterns, and mortality of the mice were monitored continuously for 14 days, during which the animals had free access to food and water.

For the sub-chronic toxicity study, mice were administered FRLE at a daily dose of 16.6 g/kg via oral gavage for 30 consecutive days. Throughout the study period, the survival status and activity levels of the mice were recorded daily while maintaining standard ad libitum feeding and watering conditions.

### 2.7. Study on Rat Serum Constituents and Their Metabolites

Twelve Sprague-Dawley rats (6 weeks old, 350 ± 10 g) were housed in a standard environment with ad libitum access to food and water. The rats were randomly divided into two groups (*n* = 6 per group): the blank group and the administration group. FRLE was dissolved in distilled water to prepare a 0.83 g/mL suspension. After 12 h of fasting (with free access to water), rats in the Administration group received a single dose of 1 mL/100 body weight via oral gavage. Blood samples (≈ 0.3 mL) were collected from the retro-orbital venous plexus at 0 (pre-dose), 0.083, 0.25, 0.5, 0.75, 1, 1.5, 2, 3, 4, 6, 8, 12, 24, 36, and 48 h post-administration into heparinized tubes. Water was restricted for 2 h, and food was withheld for 4 h post-dosing. Serum was separated by centrifugation at 3000 rpm for 15 min and stored for analysis.

For protein precipitation, 0.1 mL of serum was mixed with 0.3 mL of acetonitrile containing 0.1% formic acid (*v*/*v*) and vortexed for 5 min. The mixture was centrifuged at 13,780 *g* for 10 min at 4 °C. The supernatant was transferred and evaporated to dryness under nitrogen at 37 °C. The residue was reconstituted in 0.1 mL of methanol, vortexed, and centrifuged again at 13,780 *g* for 10 min at 4 °C to obtain the final plasma samples.

Reference standard stock solutions (1 mg/mL) were prepared in methanol. A mixed standard solution (50 μg/mL) was created by diluting the stocks, and 10 μL of this mixture was spiked into 1 mL of blank serum to prepare the quality control (QC) plasma samples. Samples were analyzed using TOF-MRM mode, and absorbed constituents were identified based on retention time (RT) and characteristic fragment ions.

Metabolic pathways were predicted using BioTransformer 3.0 (https://biotransformer.ca/, accessed on 31 May 2026), a machine learning-based tool [17,18]. The chemical structures of 20 identified constituents (including malic acid, quinic acid, and tiliroside, etc.) were converted into SMILES strings via ACD/ChemSketch and processed to predict Phase I and Phase II metabolites, specifically targeting cytochrome P450 enzymatic transformations. To facilitate identification, CFM-ID 4.0 (https://cfmid.wishartlab.com/, accessed on 31 May 2026) was employed to simulate the fragmentation behavior and generate theoretical MS/MS spectra for the predicted metabolites in ESI- mode [19]. Finally, the absorbed metabolites were identified by integrating Waters UNIFI 1.9.4 with the results from BioTransformer and CFM-ID. A positive identification required at least one characteristic fragment ion to match between the experimental and theoretical MS/MS spectra.

### 2.8. Mechanism Prediction via Network Pharmacology and Molecular Docking

The targets of 15 prototype constituents identified in FRLE (including rutin, pomolic acid, and euscaphic acid, etc.) were retrieved from the TCMSP database (https://www.tcmsp-e.com/tcmsp.php, accessed on 3 March 2025) [20]. For their metabolites, potential targets were predicted using the SwissTargetPrediction database (http://swisstargetprediction.ch/, accessed on 4 March 2025) [21]. All identified target names were standardized to gene symbols using the STRING database (https://cn.string-db.org/, accessed on 3 March 2025) to establish the constituent-target dataset [22]. Concurrently, the disease-target dataset was constructed by searching the DisGeNET database (https://disgenet.com/, accessed on 3 March 2025) for “hyperlipemia” (Disease ID: C0020473), with retrieved genes similarly standardized via STRING [23]. The intersection of constituent and disease targets was determined using the Venny 2.1.0 tool to identify potential therapeutic targets [24]. Finally, Kyoto Encyclopedia of Genes and Genomes (KEGG) pathway enrichment analysis was performed on these common targets using the DAVID database (https://davidbioinformatics.nih.gov/, accessed on 4 March 2025) [25,26]. Constituent–target–pathway network was visualized using Cytoscape 3.9.1 [27].

Molecular docking was conducted to evaluate the binding affinities between the identified constituents (prototypes and metabolites) and HLP-related proteins. Chemical structures were drawn using ACD/ChemSketch and converted into 3D configurations via Open Babel, followed by energy minimization using the MMFF94 force field. Ligand files were prepared using the prepare_ligand4.py script from AutoDockTools 1.5.6, which involved merging non-polar hydrogens and assigning Gasteiger charges to generate PDBQT files [28]. The 3D structures of key HLP-related receptors were retrieved from the Protein Data Bank (PDB; http://www.rcsb.org/, accessed on 5 March 2025). Receptors were processed using the prepare_receptor4.py script, including adding hydrogen, removing water molecules, and deleting heteroatoms to generate the final PDBQT files.

Molecular docking simulations were performed using AutoDock Vina 1.2.3 in an Ubuntu 22.04 LTS environment [29]. A global conformational search was executed with an exhaustiveness of 25 and a spacing of 1 Å, with other parameters set to default. The conformation with the lowest binding energy for each ligand-receptor pair was selected for further analysis.

### 2.9. Western Blotting Analysis

Mouse liver tissues (0.1 g) from the Control, Model, and FRLE treatment groups were homogenized in 1 mL of pre-cooled RIPA lysis buffer supplemented with 1% PMSF. Homogenization was performed using grinding beads at 2000 rpm for three cycles (1 min per cycle, with 1 min intervals on ice). The lysates were centrifuged at 13,780 *g* for 15 min at 4 °C, and the supernatant was collected. Total protein concentration was quantified using a BCA assay. Protein samples were then diluted with distilled water and 4× loading buffer to a final concentration of 5 μg/μL (sample to buffer ratio of 3:1, *v*/*v*). The mixtures were denatured in a metal bath at 100 °C for 10 min and cooled for subsequent use.

Equal amounts of protein (25 μg) were separated via 10% SDS-PAGE (150 V for 90 min) and transferred onto PVDF membranes using a wet transfer method (100 V for 60 min). The membranes were blocked with 5% non-fat milk at room temperature for 1 h and subsequently washed three times with TBST. The membranes were then incubated overnight at 4 °C with primary antibodies against NFE2L2 (1:2500), eNOS (1:500), CYP1A1 (1:1000), PPARG (1:1000), MAPK1 (1:10,000), and GAPDH (1:5000). After washing, the membranes were incubated with secondary antibodies (1:10,000) for 1 h at room temperature. Protein bands were visualized using an ECL chemiluminescence kit and captured with a Bio-Rad imaging system (Gel Doc XR, Bio-Rad, Berkeley, CA, USA). The gray values of the protein bands were analyzed using Image Lab 6.0 software, and the relative protein expression levels were normalized to GAPDH.

### 2.10. Ligand-Receptor Binding Mode Analysis

The ligand-receptor binding modes of the positive targets validated by Western blotting were analyzed and visualized using PyMOL (Ver. 3.0, Educational Edition) and Discovery Studio 2021 Client [30,31]. This analysis focused on characterizing the key intermolecular interactions, including hydrogen bonding, hydrophobic effects, and pi-stacking, to elucidate the molecular basis of the constituent-target recognition.

### 2.11. Statistical Analysis

Statistical analysis was performed using IBM SPSS Statistics 17.0. Data following a normal distribution were analyzed using one-way analysis of variance and presented as the mean ± standard deviation ($\bar{x}\;\pm \;s$). For post hoc multiple comparisons, LSD test was applied if variances were homogeneous, while Tamhane’s T2 test was utilized in cases of unequal variances. Data that did not conform to normal distribution were analyzed using the rank-sum test. To visualize sample clustering and assess overall variations among the groups, an unsupervised PCA was conducted. The LC-MS dataset was mean-centered and Pareto-scaled prior to PCA modeling, which was performed using Waters EZinfo 3.0 software. The significance level was set at α = 0.05, and the value of *p* < 0.05 was considered statistically significant.

## 3. Results

### 3.1. Chemical Constituent Variations in FRLE from Different Origins

**Figure 1:**
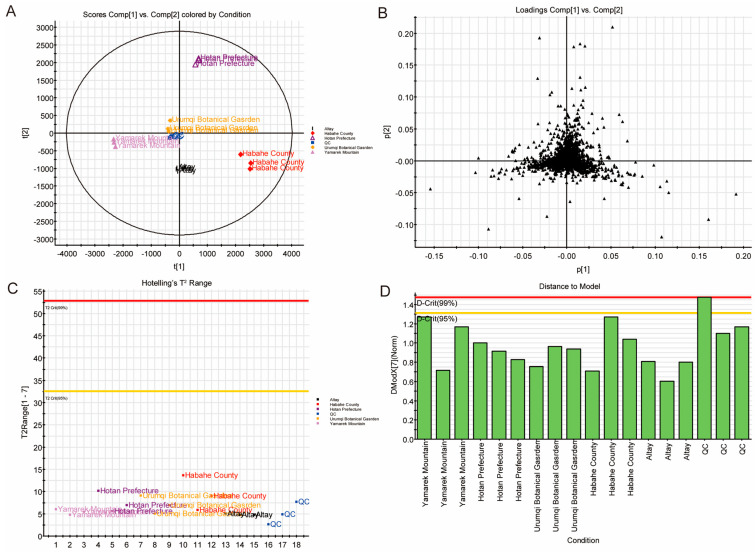
Five PCA diagrams of FRLE components from different origins. (**A**) PCA-X score plot. (**B**) loading diagram. (**C**) Hotelling T^2^ plot. (**D**) Distance to model diagram.

The data processed by Progenesis QI were imported into EZInfo V3.0 for PCA. According to the report generated, the dataset comprised 18 samples with 8507 variables and no missing values. Of these, 8501 variables were utilized for PCA. A PCA-X model was established using Pareto scaling without further data transformation. Seven principal components were extracted, cumulatively explaining 98% of the total variance. The PCA-X score plot (Figure 1A) revealed distinct clustering and segregation among the samples. Specifically, samples from Yamalike Mountain, Hotan, Habahe, and Altay exhibited significant dispersion, indicating substantial differences in their mass spectrometry-based chemical profiles.

The loading plot (Figure 1B) illustrates the distribution of the 8501 variables across the principal component axes. Several variables exhibited high loading values, suggesting they contribute significantly to the total variance and serve as key markers for differentiating the origins. Hotelling’s T^2^ and the DModX were employed as critical metrics to assess sample outliers and the goodness of fit, respectively. In this study, the Hotelling’s T^2^ plot (Figure 1C) showed no extreme outliers, confirming the high quality of data acquisition. Furthermore, the DModX analysis (Figure 1D) demonstrated that all samples—with the exception of a single QC injection that slightly exceeded the 95% confidence interval—were within acceptable limits. This indicates a robust fit between the experimental observations and the PCA model.

**Figure 2:**
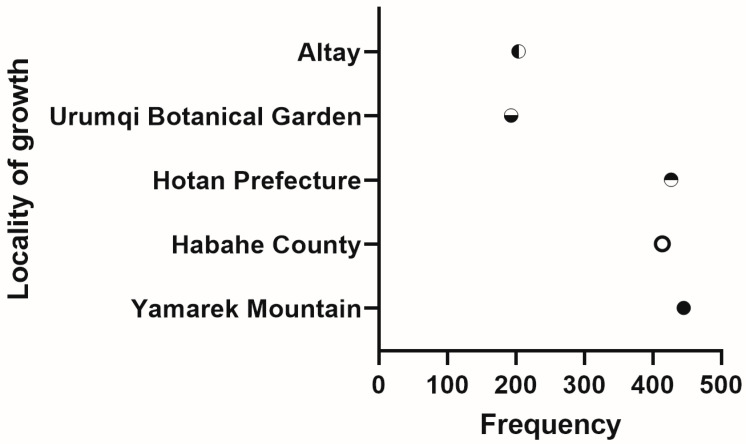
Origin Name Frequency Chart.

To further investigate inter-group variations, partial least squares discriminant analysis (PLS-DA) was performed using EZInfo V3.0, supplemented by differential constituent analysis in Progenesis QI. By applying strict filtering criteria of VIP ≥ 1 and q-value < 0.01, a total of 1736 significant variables were screened. Using mass spectrometry response intensity as the evaluation metric, we calculated the frequency of high-response compounds across the five different origins (Figure 2). The statistical analysis revealed that FRL from Yamalike Mountain possessed the greatest abundance of high-response chemical constituents. Based on these findings, the Yamalike Mountain sample was designated as the primary subject for all subsequent chemical profiling and pharmacodynamic studies.

### 3.2. Identification and Validation of Chemical Constituents in FRLE

A systematic compound identification method was established using the UNIFI informatics platform, integrated with an in-house chemical database containing 354 compounds with fragmentation data curated from literature and preliminary studies. After binary comparison and exclusion of impurities from blank solvents, the remaining data were matched based on accurate mass with a tolerance of 10 ppm and a precursor ion response threshold of 10,000. The software was configured to generate theoretical fragments from input structures and incorporate all isotopic peaks for matching. To minimize false positives, a compound was only tentatively identified if it contained at least one experimentally reported fragment from literature. The results revealed that FRLE is rich in diverse chemical classes, including organic acids, flavonoids and their glycosides, triterpenoid acids, sugars and derivatives, phenolic acids and their glycosides, iridoid glycosides, tannins, amino acids, fatty acids, polyphenols, and lactones (Table 1).

The *m*/*z* values of the compounds listed in Table 1 were imported into an inclusion list for data-dependent acquisition (DDA) mode. No exclusion list was utilized. The MS/MS trigger threshold was set at 10,000, with low and high collision energy ranges of 10–20 eV and 60–80 eV, respectively. Reference standards were procured based on these findings and injected into the mass spectrometer for collision-induced dissociation (CID) optimization (Table 2).

Finally, the reference standards and FRLE samples were analyzed using the optimized CID parameters for MS/MS data acquisition. Compounds in FRLE with retention times (RT) and fragment ions identical to those of the reference standards were confirmed as positively identified constituents (Appendix A).

### 3.3. Quantitative Determination of Chemical Constituents in FRLE

Calibration curves were established using the TOF-MRM mode. The limit of detection (LOD) and the limit of quantification (LOQ) were defined based on signal-to-noise (S/N) ratios of 3 and 10, respectively. Detailed parameters, including regression equations, correlation coefficients (R^2^), and linear ranges for each constituent, are summarized in Table 3. The contents of the identified compounds in FRLE are summarized in Table 4.

### 3.4. Pharmacodynamic Evaluation of FRLE Against HLP

**Figure 3:**
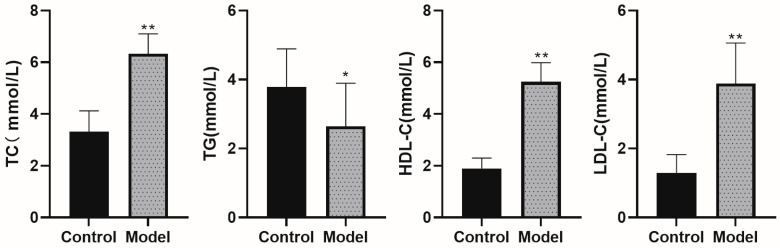
TC, TG, HDL-C, and LDL-C statistical charts (*: *p* < 0.05, **: *p* < 0.01).

The levels of TC, TG, HDL-C, and LDL-C were determined following the manufacturer’s protocols. Each sample was measured in three independent replicates, and the mean value was used for statistical analysis (Figure 3). The model group exhibited significantly higher levels of TC and LDL-C compared to the control group (*p* < 0.01), confirming the successful establishment of the HLP model.

**Figure 4:**
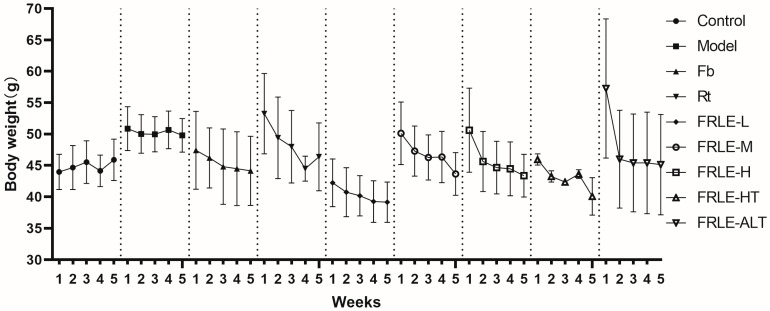
Body weight of 5 weeks statistics chart (*n* = 6).

Following successful modeling, the mean body weights of mice in each group were plotted (Figure 4). The body weights of the control group and model group remained relatively stable throughout the study. In contrast, the FRLE-ALT group showed a significant weight decrease from week 1 to week 2, followed by a plateau until week 4, and a further decline in week 5. The Rt group displayed a continuous weekly weight reduction. Other drug intervention groups generally exhibited a gradual downward trend in body weight.

**Figure 5:**
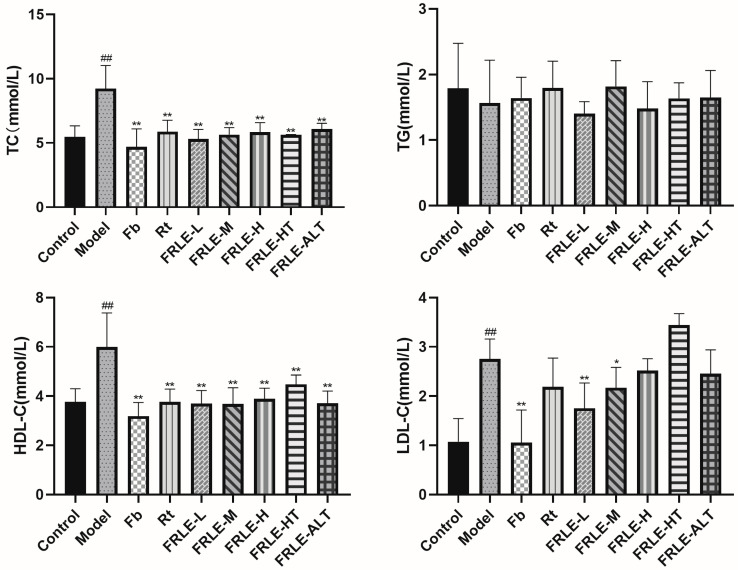
TC, TG, HDL-C, and LDL-C statistical charts (#: model group vs. control group *p* < 0.05, ##: *p* < 0.01, *: drug intervention groups vs. model group, *p* < 0.05, **: *p* < 0.01).

The serum levels of TC, TG, HDL-C, and LDL-C for all groups are summarized in Figure 5. Compared with the control group, the model group showed significantly elevated TC and LDL-C levels (*p* < 0.01), validating both the induction and maintenance of HLP. In the positive control groups, Fb significantly reduced TC and LDL-C levels (*p* < 0.01), while Rt significantly lowered TC (*p* < 0.01) compared to the model group, which is consistent with their clinical indications. These results confirm the reliability of the experimental environment and conditions. Notably, the FRLE-L, FRLE-M, FRLE-H, FRLE-HT, and FRLE-ALT groups all demonstrated a significant reduction in TC compared to the model group (*p* < 0.01); however, no clear dose-dependent relationship was observed among the three FRLE dosage levels. Regarding LDL-C, only the FRLE-L (*p* < 0.01) and FRLE-M (*p* < 0.05) groups showed significant decreases, whereas the FRLE-H and FRLE-ALT groups exhibited non-significant reductions (*p* > 0.05).

**Figure 6:**
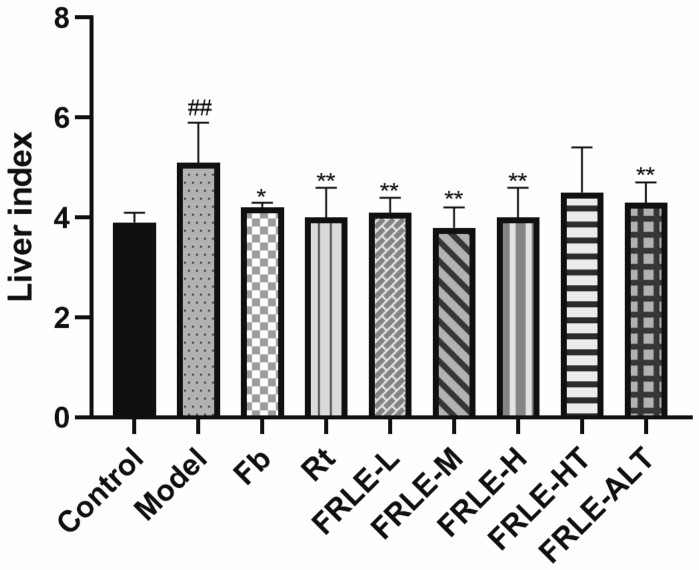
Liver index statistical charts (#: model group vs. control group *p* < 0.05, ##: *p* < 0.01, *: drug intervention groups vs. model group, *p* < 0.05, **: *p* < 0.01).

The liver index was calculated after rinsing and weighing the liver tissues (Figure 6). The model group showed a significant increase in the liver index compared to the control group. Except for the FRLE-HT group, all other drug-treated groups exhibited a significant decrease in liver index compared to the model group.

**Figure 7:**
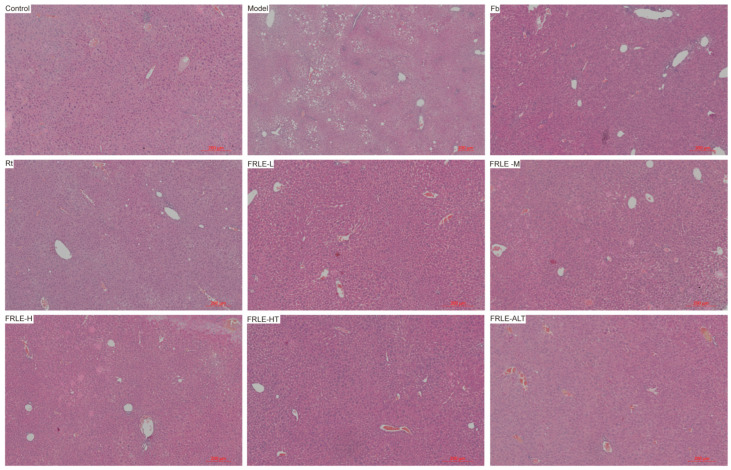
H&E staining results of liver. All images were recorded at a magnification of 200×. (Note: All panels represent standard H&E staining. Minor variations in background coloration among image panels are due to light intensity and white balance fluctuations during brightfield microscopy acquisition and do not reflect differences in staining protocols.)

The H&E staining results of liver tissues are presented in Figure 7. In the model group, a large number of large, rounded vacuoles were observed in the field of view compared to the control group, indicating the occurrence of hepatic steatosis. Compared to the model group, the Fb and Rt groups showed a reduction in vacuoles, suggesting an improvement in hepatic steatosis. Similarly, the FRLE-L, FRLE-M, FRLE-H, FRLE-HT, and FRLE-ALT groups all exhibited fewer vacuoles, demonstrating the efficacy of FRLE in alleviating hepatic steatosis.

### 3.5. Safety Evaluation of FRLE

In the acute toxicity study, FRLE was administered via oral gavage at a concentration of 830 mg/mL (0.2 mL/10 g body weight). The treatment was repeated three times at 4 h intervals. The clinical signs, dietary intake, and mortality of the mice were monitored for 14 consecutive days. No mortality was observed throughout the observation period. On the first day, a slight decrease in activity and dietary intake was noted, which was attributed to the stress of repeated gavaging and the large volume administered, leading to temporary satiety. However, from days 2 to 3, the mice exhibited a gradual recovery in activity, including increased locomotor behavior within the cages and normalized food and water consumption. From day 4 to 14, the mice fully regained their baseline physiological state, exhibiting active climbing and normal metabolic frequency. These results indicate that FRLE possesses no significant acute toxicity (Table 5).

The sub-chronic toxicity study was conducted using the same dosage, with daily oral gavage for 30 consecutive days. Body weights were recorded weekly to adjust the dosage volume, and parameters including mortality, clinical signs, food/water intake, and fur condition were monitored daily. Throughout the 30-day cycle, all mice survived healthily, except for two accidental deaths attributed to technical errors during the gavage procedure rather than drug toxicity. Activity levels and dietary intake returned to normal within 4 h post-administration each day. Furthermore, no abnormalities in fur color (e.g., yellowing or dullness) were observed during the study. These findings suggest that FRLE exhibits no significant cumulative toxicity under the tested conditions.

### 3.6. Identification of Absorbed Constituents and Their Metabolites in Rat Plasma

By comparing the MS profiles of drug-containing plasma with blank plasma and reference standards, 15 prototype constituents were identified as absorbed components: rutin, pomolic acid, euscaphic acid, hyperoside, isoquercitrin, kaempferol, kaempferol-3-O-rutinoside, tartaric acid, citric acid, malic acid, quinic acid, tiliroside, astragalin, ellagic acid, and oleanolic acid (Appendix B).

Integrating Waters UNIFI 1.9.4 with BioTransformer 3.0 and CFM-ID 4.0, a total of 14 metabolites (M1–M14) were characterized in rat plasma (Appendix C). The detailed metabolic transformations are described as follows:

M1 (*m*/*z* 285.0455, [M-H]^−^): A dehydroxylation product of quercetin. Its fragment ions at *m*/*z* 151.0030 and 255.0298 were consistent with the predicted MS/MS spectra.

M2 (*m*/*z* 303.0529, [M-H]^−^): Formed via carbonyl reduction of quercetin. The fragment ion at *m*/*z* 285.0405 matched the predicted data.

M3 (*m*/*z* 477.0673, [M-H]^−^): A glucuronidated product of quercetin catalyzed by UDP-glucuronosyltransferase (UGT). The fragment at *m*/*z* 255.0292 was consistent with predictions.

M4 (*m*/*z* 228.9641, [M-H]^−^): A secondary alcohol sulfation product of tartaric acid mediated by alcohol sulfotransferase. The fragment at *m*/*z* 136.9387 matched the prediction.

M5 (*m*/*z* 125.0242, [M-H]^−^): Formed via decarboxylation and N-glucuronidation of gallic acid. Due to low collision energy, no diagnostic ring-cleavage fragments were observed.

M6 (*m*/*z* 345.0437, [M-H]^−^): An aromatic O-glucuronide of gallic acid. Fragments at *m*/*z* 169.0124 and 123.0086 were consistent with predicted results.

M7 (*m*/*z* 309.0444, [M-H]^−^): An alkyl-OH-glucuronide or fatty acid O-glucoside of malic acid. Characteristic ions at *m*/*z* 193.0354, 193.0154, 147.0299, and 87.0088 were identified.

M8 (*m*/*z* 276.1085, [M-H]^−^): A carnitine conjugate of malic acid mediated by carnitine O-acetyltransferase. Fragments at *m*/*z* 217.0302, 173.0567, and 87.0088 matched the predictions.

M9 (*m*/*z* 477.0312, [M-H]^−^): An aromatic O-glucuronide of ellagic acid. Fragments at *m*/*z* 300.9947 and 270.9904 were consistent with the simulated spectra.

M10 (*m*/*z* 301.0362, [M-H]^−^): An ortho-hydroxylation product of kaempferol mediated by CYP1A2. Fragments at *m*/*z* 257.0765 and 255.0881 matched the predictions.

M11 (*m*/*z* 461.0729, [M-H]^−^): An aromatic O-glucuronide of kaempferol. Fragments at *m*/*z* 255.0297 and 151.0031 were consistent with predicted data.

M12 (*m*/*z* 663.3758, [M-H]^−^): An alkyl-OH-glucuronide of euscaphic acid. Diagnostic fragments were found at *m*/*z* 645.3051, 487.3424, and 193.0345.

M13 (*m*/*z* 313.0137, [M-H]^−^): Derived from aromatic L-amino acid decarboxylation and glucuronidation of protocatechuic aldehyde. Fragments at *m*/*z* 175.0238, 137.0242, and 109.0292 matched the predictions.

M14 (*m*/*z* 527.0504, [M-H]^−^): A primary alcohol sulfation product of astragalin. Characteristic fragments at *m*/*z* 497.0737 and 151.0034 were observed.

### 3.7. Potential Mechanism of FRLE Against HLP Predicted by Network Pharmacology and Molecular Docking

**Figure 8:**
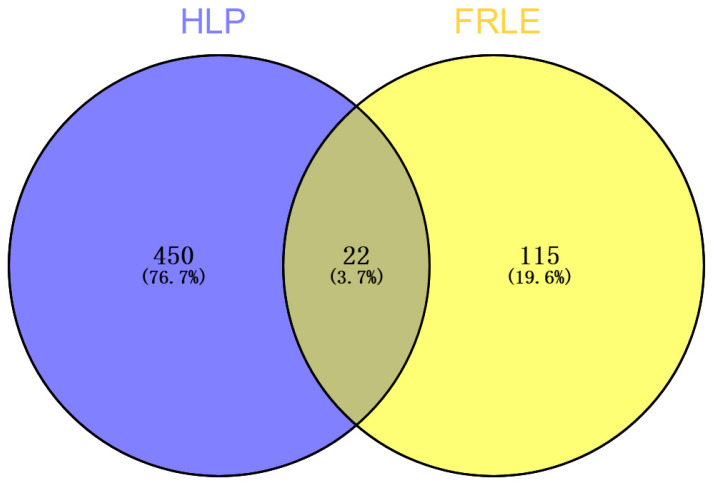
Common targets of HLP and FRLE targets.

The disease-target dataset for HLP comprised 472 targets, while the constituent-target dataset for FRLE included 137 targets. Using the Venny 2.1.0 tool, 22 overlapping targets were identified as the core therapeutic candidates (Figure 8). These intersection targets included PPARA, HSD11B1, ABCB1, CYP19A1, PYGL, TNF, PPARG, ACHE, MPO, SELP, NR1H4, ESR1, PTGS2, CASP1, ESRRA, AHR, ESR2, ALOX5, LTB4R, CYP2D6, SERPINB2, and SERPIND1.

**Figure 9:**
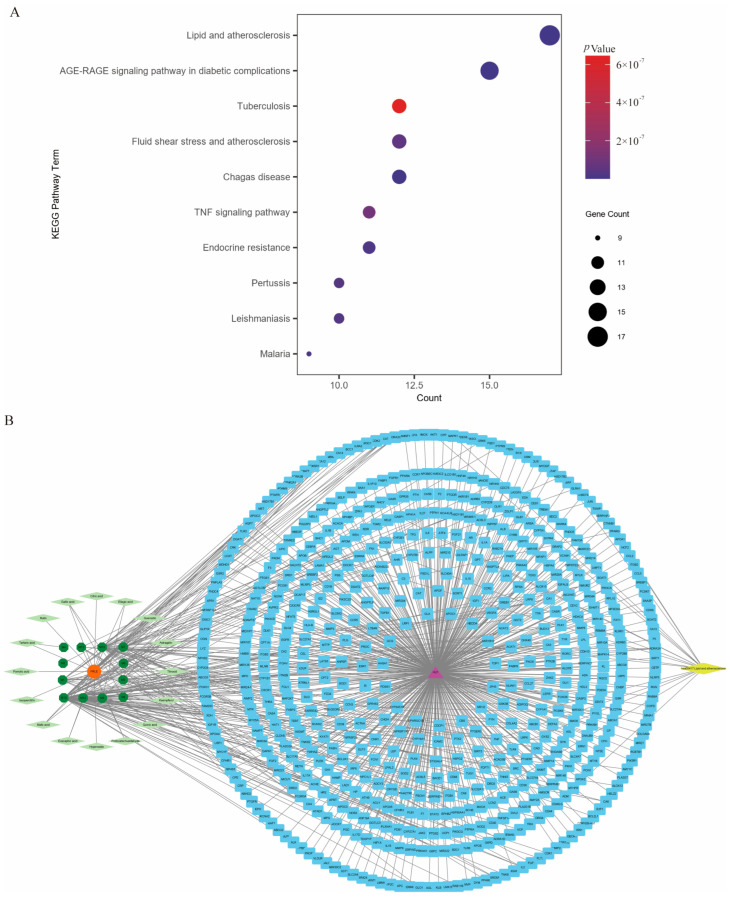
Network pharmacology analysis of FRLE in the treatment of HLP. (**A**) Top 10 KEGG pathways enrichment. (**B**) Drug-component-disease-target-pathway network diagram.

KEGG pathway enrichment analysis was performed on these common targets. The pathway with the lowest *p*-value (FDR < 0.01) and highest count, “Lipid and atherosclerosis” (hsa05417), was identified as the primary therapeutic pathway (Figure 9A). Further analysis revealed 17 common targets between the identified intersection targets and the genes involved in this specific pathway, namely VCAM1, NOS3, MMP3, MAPK14, TNF, MMP9, IL6, CD40LG, IL1B, CASP1, CYP1A1, BAX, CCL2, AKT1, MAPK1, PPARG, and NFE2L2. Construct the constituent-target-pathway diagram (Figure 9B).

**Figure 10:**
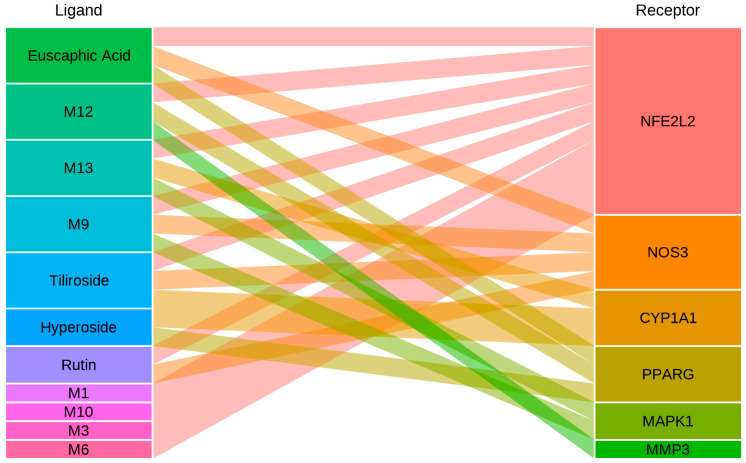
Results of molecular docking results with binding energy ≤ −9 kcal/mol.

Molecular docking was employed to evaluate the binding affinities between the constituents and the key targets (Table 6). The docking results were ranked by binding energy, and interactions with energy values ≤ −9 kcal/mol were prioritized. Based on the frequency of occurrence among high-affinity ligand-receptor pairs, the top five targets—NFE2L2, NOS3, CYP1A1, PPARG, and MAPK1—were selected as the key candidates for subsequent Western blot validation. Detailed docking parameters and visual configurations are summarized in Table 7 and Figure 10.

### 3.8. Western Blotting Analysis Results

**Figure 11:**
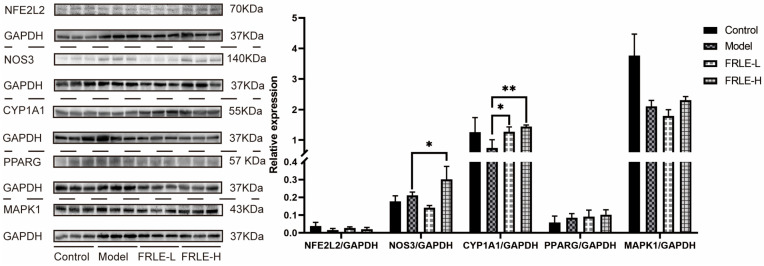
Western blot results (*n* = 3, *: FRLE-L group and FRLE-H group vs. model group, *p* < 0.05, **: *p* < 0.01).

The results of the Western blotting analysis are illustrated in Figure 11. Compared to the model group, although NFE2L2 expression showed a slight upward trend in both the FRLE-L and FRLE-H groups, the differences did not reach statistical significance (*p* > 0.05), suggesting that further validation with larger sample sizes may be warranted. For NOS3 (eNOS), a marginal, non-significant decrease in expression was observed in the FRLE-L group (*p* > 0.05), whereas a significant up-regulation was detected in the FRLE-H group (*p* < 0.05). Regarding CYP1A1, both the FRLE-L and FRLE-H groups exhibited significantly elevated expression levels compared to the model group (*p* < 0.05). Similarly, the expression of PPARG showed an upward trend in both FRLE-treated groups, yet no statistically significant difference was established (*p* > 0.05). Finally, for MAPK1, a minor decrease in expression was noted in the FRLE-L group while an increase was found in the FRLE-H group; however, neither change was statistically significant (*p* > 0.05), indicating these variations might represent non-significant fluctuations rather than definitive treatment effects.

### 3.9. Analysis of Ligand-Receptor Binding Modes

**Figure 12:**
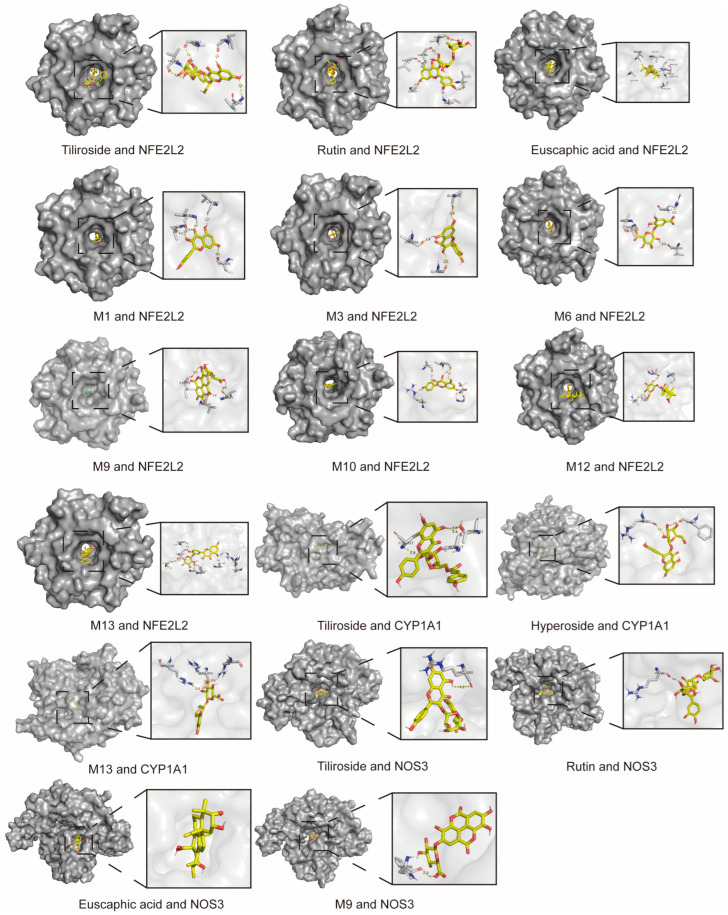
Analysis of ligand-receptor binding mode.

Molecular docking simulations revealed that euscaphic acid, tiliroside, hyperoside, rutin, and metabolites M1, M3, M6, M9, M10, M12, and M13 successfully docked into the active pockets of NFE2L2, NOS3, CYP1A1, and PPARG. The binding stability was primarily mediated by diverse intermolecular forces, including hydrogen bonding, π-alkyl interactions, π-sigma interactions, π-π stacking, and π-anion interactions. These interactions facilitate robust ligand-receptor recognition and binding, providing a structural basis for their biological activities (Figure 12).

## 4. Discussion

This study confirms that the types and levels of secondary metabolites in FRL are closely related to their growth environments. UPLC-QTOF-MS analysis demonstrated that FRL from Yamalike Mountain possesses the greatest chemical diversity, significantly surpassing samples from the Botanical Garden. This variation likely stems from the semi-alpine habitat of Yamalike Mountain, where the substantial diurnal temperature range promotes the accumulation of secondary metabolites [32,33,34,35]. In contrast, excessive anthropogenic interventions in the Botanical Garden, such as pruning and intensive irrigation, may lead to the dilution or loss of specific constituents [36,37,38]. It is worth noting that the selection of Yamalike Mountain samples as the primary material for downstream pharmacological experiments was an analytical choice driven by chemical diversity and marker abundance, rather than a pre-demonstrated superiority in biological efficacy. Because the Yamalike Mountain samples possessed the most diverse array and the highest abundance of high-response differential constituents, utilizing this origin maximized our capacity to qualitatively and quantitatively characterize the comprehensive chemical profile of FRLE via UPLC-QTOF-MS. Characterizing such a highly enriched chemical repertoire is a crucial prerequisite for network pharmacology, ensuring that a wider spectrum of potential bioactive components can be captured and evaluated during subsequent target prediction and mechanism elucidation.

The complexity of TCM, characterized by numerous isomers, poses a risk of false positives when relying solely on MS spectral matching. To address this, we established an in-house library integrating RT of reference standards and employed a dual-verification strategy using MS^E^ and DDA modes. This approach overcomes the limitations of RT variability and significantly enhances the accuracy of isomer identification, providing a high-fidelity chemical foundation for studying the pharmacodynamic basis of FRLE.

In conventional HLP models, TG levels typically increase while HDL-C levels decrease. However, our model group exhibited unexpected trends. The observed decrease in TG may be attributed to an increase in intrahepatic TG synthesis, which consequently lowers peripheral blood TG levels [39]. Furthermore, while HDL-C is traditionally viewed as “good cholesterol”, recent studies suggest that excessively high levels do not necessarily confer cardiovascular protection [40,41,42]. In this study, FRLE intervention maintained HDL-C at levels consistent with the control group. This indicates that FRLE exerts a cardiovascular protective effect by normalizing or correcting abnormally elevated HDL-C levels. Ultimately, the absence of significant differences in TG and HDL-C between the treatment groups and the control group suggests that FRLE effectively maintains these parameters within a normal physiological range.

PCA revealed significant chemical variations among FRL from Yamalike Mountain, Hotan, and Altay; however, all three samples significantly reduced TC levels in HLP mice, suggesting that common constituents drive the cholesterol-lowering effect. The pharmacodynamic distinction lies in the lack of LDL-C-lowering efficacy in the Hotan sample, whereas Altay and Yamalike Mountain samples showed similar performance in reducing LDL-C and maintaining HDL-C. Therefore, FRL samples with similar primary chemical profiles can be considered viable candidates for anti-HLP drug development.

Using the UPLC-QTOF-MRM method, 20 constituents were quantified in FRLE, and 15 bioactive components were confirmed in rat plasma. These included flavonoid glycosides (rutin, tiliroside, astragalin, hyperoside, isoquercitrin, kaempferol, and kaempferol-3-O-rutinoside), triterpenoid acids (pomolic acid, euscaphic acid, and oleanolic acid), and organic acids (tartaric, citric, malic, quinic, and ellagic acid). Flavonoids are well-documented for their anti-HLP activities, while triterpenoid acids exert anti-HLP effects by inhibiting bile acid transporter [43,44,45,46]. These components constitute the material basis for the therapeutic efficacy of FRLE. Arginine was undetected in plasma, likely because as an essential amino acid, it was rapidly utilized in physiological processes post-absorption. Other components, such as gallic acid, quercetin, and protocatechuic aldehyde, were undetected due to their low initial content and subsequent dilution or rapid metabolism in vivo.

Network pharmacology and molecular docking predicted that the anti-HLP mechanism of FRLE involves 17 key targets (e.g., VCAM1, NOS3, CYP1A1, PPARG, and NFE2L2) within the Lipid and Atherosclerosis pathway (hsa05417). These targets are intricately linked to lipid imbalance, inflammation, oxidative stress, and endothelial dysfunction. Western blot validation further confirmed that the absorbed constituents and their metabolites exert anti-HLP effects by modulating NFE2L2, CYP1A1, NOS3, and MAPK1. Further research is warranted to elucidate the deeper molecular mechanisms involved [47,48,49,50,51].

Finally, a limitation of this study is the relatively small sample size (*n* = 3) utilized for the Western blotting verification. While *n* = 3 is a standard convention for preliminary screening in pharmacodynamic studies, it inherently possesses low statistical power. This limitation may account for the observed but statistically non-significant (*p* > 0.05) regulatory trends in certain protein targets, such as NFE2L2, PPARG, and MAPK1. Future studies with a larger cohort are warranted to comprehensively validate these subtle molecular alterations.

## 5. Conclusions

In summary, the chemical constituents of FRLE were qualitatively and quantitatively characterized using UPLC-QTOF-MS. Sixty compounds were identified through database matching, 20 of which were further confirmed and quantified using reference standards. PCA revealed significant chemical variations among FRL from different geographical origins, with the Yamalike Mountain sample possessing the most diverse array of differential constituents. Safety evaluations classified FRLE as a low-toxicity product, suitable for potential long-term administration. Pharmacodynamic results demonstrated that FRLE effectively ameliorates TC, HDL-C, and LDL-C levels in HLP mice, exhibiting potent anti-HLP efficacy. Mechanistically, integration of computational predictions and empirical evidence suggests that the absorbed constituents and their metabolites may exert these therapeutic effects by modulating key potential targets, including CYP1A1, NOS3, NFE2L2, and MAPK1. These findings provide a scientific basis for the development of FRLE as a safe and effective candidate for the long-term management of hyperlipidemia.

## Figures and Tables

**Table 1:** Comparison table of UNIFI chemical library.

No.	Component	Formula	Response	Adduct	Mass Error (ppm)	Observed *m*/*z*
1	Citric acid	C_6_H_8_O_7_	318,933	-H	2.6	191.0202
2	Quercetin-3-O-glucuronide	C_21_H_18_O_13_	262,295	-H	5.0	477.0698
3	Sucrose	C_12_H_22_O_11_	186,653	+HCOO, -H	3.4	387.1157
4	1-Galloyl-β-D-glucose (Glucogallin)	C_13_H_16_O_10_	176,919	-H	4.6	331.0686
5	Cynaroside (Luteolin-7-O-glucoside)	C_21_H_20_O_11_	124,064	-H	2.5	447.0944
6	Raffinose	C_18_H_32_O_16_	92,953	+HCOO, -H	2.9	549.1689
7	Isorosmarinic acid glucoside	C_24_H_26_O_13_	76,117	-H	3.5	521.1319
8	Malic acid *	C_4_H_6_O_5_	73,907	-H	5.1	133.0149
9	Mulberroside A	C_26_H_32_O_14_	67,657	-H	4.2	567.1743
10	Methyl przewalskinate	C_18_H_14_O_8_	62,415	-H, +HCOO	−1.9	357.0609
11	Quinic acid *	C_7_H_12_O_6_	61,245	-H	5.2	191.0571
12	Kaempferol-3-O-rutinoside (Nicotiflorin) *	C_27_H_30_O_15_	61,173	-H	4.1	593.1536
13	Madecassic acid	C_30_H_48_O_6_	60,051	-H, +HCOO	4.5	503.3401
14	Dihydroclovamide	C_21_H_22_O_11_	59,299	-H	4.4	449.1109
15	(-)-Epicatechin *	C_15_H_14_O_6_	57,976	-H	5.4	289.0733
16	Procyanidin C1	C_45_H_38_O_18_	55,358	-H	3.5	865.2016
17	p-Coumaric acid 4-O-glucoside	C_15_H_18_O_8_	54,587	-H	5.1	325.0946
18	Ellagic acid *	C_14_H_6_O_8_	50,846	-H	−7.3	300.9968
19	3-Hydroxy-2-pyrone	C_5_H_4_O_3_	49,801	-H	5.9	111.0094
20	Citric acid *	C_6_H_10_O_8_	46,626	-H	2.6	209.0308
21	Tiliroside *	C_30_H_26_O_13_	45,613	-H	4.4	593.1327
22	L-Arginine *	C_6_H_14_N_4_O_2_	45,197	-H	2.3	173.1048
23	Afzelin (Kaempferol-3-O-rhamnoside)	C_21_H_20_O_10_	44,536	+HCOO	5.0	477.1062
24	Rutin *	C_27_H_30_O_16_	43,272	-H	5.1	609.1492
25	Quercetin *	C_15_H_10_O_7_	42,417	-H	1.3	301.0358
26	Euscaphic acid *	C_30_H_48_O_5_	42,141	-H	−2.4	487.3417
27	Asiatic acid	C_30_H_48_O_5_	42,141	-H	−2.4	487.3417
28	Kaempferol *	C_15_H_10_O_6_	40,832	-H	3.9	285.0416
29	Homoplantaginin	C_22_H_22_O_11_	39,771	+HCOO	4.3	507.1166
30	Arabic acid	C_5_H_10_O_6_	39,411	-H	6.1	165.0415
31	Corilagin	C_27_H_22_O_18_	36,696	-H	5.2	633.0767
32	Protocatechuic aldehyde *	C_7_H_6_O_3_	33,676	+HCOO, -H	2.6	183.0304
33	Astragalin (Kaempferol-3-O-glucoside) *	C_21_H_20_O_11_	32,084	-H	−0.7	447.0930
34	Arachidonic acid	C_20_H_32_O_2_	30,255	+HCOO	9.1	349.2416
35	Oleanolic acid *	C_30_H_48_O_3_	30,151	-H	0.9	455.3535
36	Ursolic acid	C_30_H_48_O_3_	30,151	-H	0.9	455.3535
37	Pomolic acid *	C_30_H_48_O_4_	30,142	-H	3.6	471.3497
38	Eriocitrin	C_27_H_32_O_15_	29,752	-H	2.4	595.1683
39	Tartaric acid *	C_4_H_6_O_6_	26,570	-H	4.5	149.0098
40	Aromadendrin	C_15_H_12_O_6_	24,413	+HCOO	−4.3	333.0602
41	Isolicuritoside	C_26_H_30_O_13_	23,527	-H, +HCOO	3.4	549.1632
42	Quercetin-3-O-glucoside (Isoquercitrin) *	C_21_H_20_O_12_	22,946	-H	3.7	463.0899
43	L-Tryptophan	C_11_H_12_N_2_O_2_	21,270	-H	1.7	203.0830
44	Isoshaftoside	C_26_H_28_O_14_	18,467	+HCOO	−4.4	609.1434
45	Baicalein	C_15_H_10_O_5_	18,211	-H	4.7	269.0468
46	Wogonoside	C_22_H_20_O_11_	18,001	-H	4.9	459.0955
47	Dihydromorin	C_15_H_12_O_7_	17,924	-H	4.6	303.0524
48	Tormentic acid	C_30_H_48_O_5_	17,479	-H, +HCOO	3.5	487.3446
49	3-Methylfuran-2-carboxylic acid	C_6_H_6_O_3_	17,269	-H	5.3	125.0251
50	Hippophaeone	C_27_H_26_O_17_	16,654	-H	2.1	621.1110
51	Gluconolactone	C_6_H_10_O_6_	16,409	+HCOO, -H	2.8	223.0466
52	Plantamajoside	C_21_H_22_O_12_	16,361	-H	4.6	465.1060
53	Dihydromyricetin	C_15_H_12_O_8_	16,351	+HCOO	−2.5	365.0505
54	Galacturonic acid	C_18_H_26_O_19_	15,875	-H	3.0	545.1012
55	Hyperoside *	C_21_H_12_	15,153	-H, +HCOO	4.7	463.0904
56	Neogeniposide	C_16_H_22_O_10_	12,229	+HCOO	1.7	419.1202
57	Eriodictyol	C_15_H_12_O_6_	11,835	+HCOO	9.3	333.0647
58	Genistin	C_21_H_20_O_10_	11,627	+HCOO	6.6	477.1070
59	Neoeriocitrin	C_27_H_32_O_15_	11,442	-H	3.1	595.1687
60	Narcissin	C_28_H_32_O_16_	10,965	+HCOO	−7.7	669.1621

Notes: Compounds identified through retention time and MS/MS verification (*); The others are compounds that were initially labeled through database matching.

**Table 2:** Reference Product Ions and CID.

No.	Compound	CID (eV)	Parent Ion and MS/MS Fragments *m*/*z*
1	Malic acid	12	133.0148, 155.0043, 89.0252, 72.993, 171.0156
2	Quinic acid	25	191.0608, 127.0419, 109.0315, 93.0364, 87.0099, 85.0308, 81.0360, 71.0156, 59.0145
3	Tartaric acid	16	149.0065, 105.0190, 103.0037, 87.0098, 75.0083, 72.9931, 59.1045
4	Citric acid	12	191.0240, 129.0230, 111.0120, 87.0099, 85.0308
5	Tiliroside	30	593.1327, 447.0930, 285.0416
6	Ellagic acid	40	300.9901, 299.9921, 283.9984, 270.9887, 254.9337, 243.9999, 228.0052, 216.0059, 200.0129, 189.0182, 184.0178, 172.0168, 161.0253, 145.0309, 133.0307, 129.0342, 117.0354, 105.0358, 101.0408, 89.0406
7	Ursolic acid	50	455.3535, 407.3296, 391.3015, 373.2534
8	Astragalin	26	447.0930, 284.0330, 255.0296, 227.0353
9	Gallic acid	15	169.0138, 125.0253
10	Pomolic acid	40	471.3497, 453.3369, 451.3204, 411.3277, 407.3337, 405.3152, 391.3056, 375.2699
11	Euscaphic acid	40	487.3417, 469.3334, 443.3554, 425.3459, 407.3296, 393.3174, 377.2838
12	Rutin	33	609.1439, 300.0276
13	Quercetin	22	301.0358, 273.0422, 178.9995, 151.0025, 121.0300, 107.0136
14	Hyperoside	24	463.0901, 300.0276
15	Kaempferol	35	285.0416, 267.0322, 255.0296, 239.0353, 227.0353, 211.0417, 201.0569, 187.0386, 183.0440, 171.0459, 187.0495, 183.0440, 171.0459, 167.0495, 159.0461, 143.0500, 131.0495, 117.0354, 108.0200, 93.0340, 83.0130, 77.0402, 65.0034
16	Kaempferol-3-O-rutinoside	31	593.1526, 285.0416
17	Raffinose	25	503.1721, 323.1029, 281.1136, 221.0937, 179.0842, 161.0734, 143.0626, 119.0625, 113.0519, 101.0527, 89.0508, 87.0354
18	Corilagin	30	633.1049, 463.0744, 301.0135, 275.0308, 273.0172
19	Sucrose	21	341.1320, 179.0842, 161.0734, 149.0759, 143.0626, 119.0625, 113.0519, 101.0527, 89.0508, 71.0400, 59.0377
20	L-Arginine	15	173.1086, 156.0793
21	Oleanolic acid	50	455.3352, 407.3146
22	Tormentic acid	40	487.3449, 469.3334, 467.3215, 441.3373, 437.3073, 427.3235, 423.3285, 405.3196, 393.3186, 377.2861, 369.2828
23	Asiatic acid	40	487.3692, 469.3565, 441.3586, 427.3438, 423.3486, 407.3488, 393.3367, 391.3168, 373.2727
24	Cynaroside	20	477.1004, 285.0450
25	(-)-Epicatechin	40	289.0740, 245.0819, 203.0699, 179.0349, 151.0388, 137.0252, 109.0295
26	Isoquercitrin	25	463.0872, 300.0268, 271.0241, 255.0291, 243.0294, 227.0341, 151.0030
27	Protocatechuic aldehyde	25	137.0282, 119.0218, 108.0239, 92.0289, 79.9588, 63.9638

**Table 3:** Linear equation and LOD and LOQ.

Compound	Linear Equation	R^2^	LOD	LOQ
Ellagic acid	y = 37,095.0x − 26,216.0	0.9965	0.44	1.32
L-Arginine	y = 6012.8x + 2865.0	0.9997	0.07	0.23
Tartaric acid *	y = 13,874.0x − 50.4	0.9978	17.00	59.00
Gallic acid	y = 54,065.0x + 4319.1	0.9946	0.04	0.15
Quercetin *	y = 586,216.0x − 138,680.0	0.9988	28.00	93.00
Citric acid	y = 14,931.0x − 25,186.0	0.9967	0.17	0.57
Pomolic acid	y = 10,536.0x + 3085.1	0.9987	0.40	1.32
Astragalin	y = 476.8x − 4691.2	0.9996	6.43	21.43
Oleanolic acid	y = 615.9x − 2360.9	0.9992	1.19	3.96
Kaempferol *	y = 111.0x − 1551.6	0.9988	6.00	19.00
Kaempferol-3-O-rutinoside *	y = 146.5x − 6074.7	0.9979	12.00	39.00
Protocatechuic aldehyde *	y = 54.2x − 1110.9	0.9966	7.00	23.00
Rutin *	y = 1461.0x + 262.9	0.9987	0.10	0.03
Hyperoside *	y = 0.61772.0x + 3.2	0.9915	0.46	1.50
Euscaphic acid *	y = 36.2x + 68,207.9	0.9923	6.46	21.50
Malic acid	y = 1.0x + 166.1	0.9942	0.14	0.49
Isoquercitrin *	y = 72.0x + 1196.5	0.9984	0.40	1.30
Quinic acid *	y = 4.7x + 33.7	0.9979	3.70	12.50
Tiliroside *	y = 0.4x + 3.8	0.9913	2.00	6.60
(-)-Epicatechin *	y = 107,257.0x + 4224.2	0.9977	21.00	71.00

Notes: Concentration units are expressed as ng/mL for compounds marked with an asterisk (*); otherwise, units are μg/mL.

**Table 4:** Contents of major chemical constituents in FRLE.

No.	Compound	Constituent (mg/g)
1	Ellagic acid	0.7308
2	Tartaric acid	0.0779
3	Citric acid	1.9809
4	Astragalin	11.0205
5	Gallic acid	0.1751
6	Pomolic acid	1.0536
7	Quercetin	0.0289
8	Kaempferol	0.0143
9	Kaempferol-3-O-rutinoside	0.0175
10	L-Arginine	0.4624
11	Oleanolic acid	17.3533
12	Protocatechuic aldehyde	0.0034
13	(-)-Epicatechin	0.0364
14	Rutin	0.0182
15	Hyperoside	0.5964
16	Euscaphic acid	4.8906
17	Malic acid	6.5154
18	Isoquercitrin	1.6794
19	Quinic acid	1.3046
20	Tiliroside	0.5184

**Table 5:** The number of mouse deaths, vitality, bite and sup over a 14-day period (*n* = 20).

Status Check Item	Number of Days													
	1	2	3	4	5	6	7	8	9	10	11	12	13	14
The number of deaths	0	0	0	0	0	0	0	0	0	0	0	0	0	0
Vitality	−	+	+	++	++	++	++	++	++	++	++	++	++	++
food and water intake	−	+	+	++	++	++	++	++	++	++	++	++	++	++

Note: −, diminished vitality with markedly reduced food and water intake; +, moderate vitality, food, and water intake; ++, vigorous vitality with normal food and water intake.

**Table 6:** Detailed grid box parameters and coordinates for molecular docking receptors.

Protein	PDBID	Center_x	Center_y	Center_z	Size_x	Size_y	Size_z
VCAM1	1VCA	12	41.24	60.5	32	30	89
NOS3	1M9J	10.8	9.42	51	54	68	49
MMP3	1B3D	3	20.2	32.5	39	37	39
APK14	1BL6	1	19	37	53	55	54
TNF	1A8M	26	60	45.2	47	40	41
MMP9	1GKC	61	22.22	114	36	39	40
IL6	4O9H	−37	23	12	34	37	43
D40LG	3LKJ	12	16	11	43	31	32
IL1B	1HIB	23.5	2	76	39.5	39	34
CASP1	1BMQ	47.45	64.5	−1	53	38	62
YP1A1	4I8V	−19	37	−39	62	50	59
BAX	4BDU	18	102	85	45	51	76
CCL2	1DOK	7	44	33	39	25	34
AKT1	1H10	22	15.5	10	38	32.2	39
MAPK1	1PME	−3	7	45	57	41	61
PPARG	1FM6	21	−20	2	42	47	57
NFE2L2	2FLU	17.57	19	9	46	46	45

**Table 7:** Molecular docking results.

Receptor-Ligand Name	Binding Energy (kcal/mol)
NFE2L2_M9	−10.832
NFE2L2_Euscaphic acid	−10.824
NFE2L2_M13	−10.583
CYP1A1_M13	−10.174
NFE2L2_M12	−10.024
MMP3_M12	−10.017
PPARG_M12	−9.879
NFE2L2_M10	−9.871
CYP1A1_Tiliroside	−9.804
NOS3_Rutin	−9.75
CYP1A1_Hyperoside	−9.681
NFE2L2_M3	−9.669
NFE2L2_Rutin	−9.574
NFE2L2_M6	−9.405
MAPK1_M9	−9.387
NFE2L2_M1	−9.376
PPARG_Hyperoside	−9.299
NOS3_Tiliroside	−9.168
NOS3_Euscaphic acid	−9.13
PPARG_Euscaphic acid	−9.127
NOS3_M9	−9.125
MAPK1_M13	−9.051
NFE2L2_Tiliroside	−9.027

**Figure A1:**
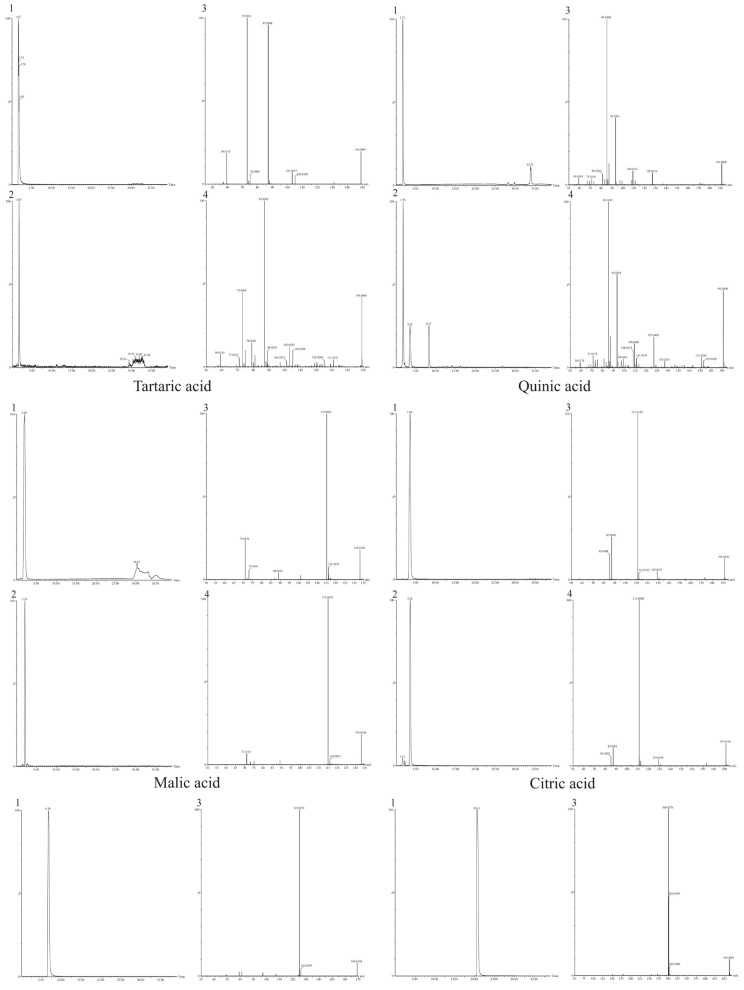

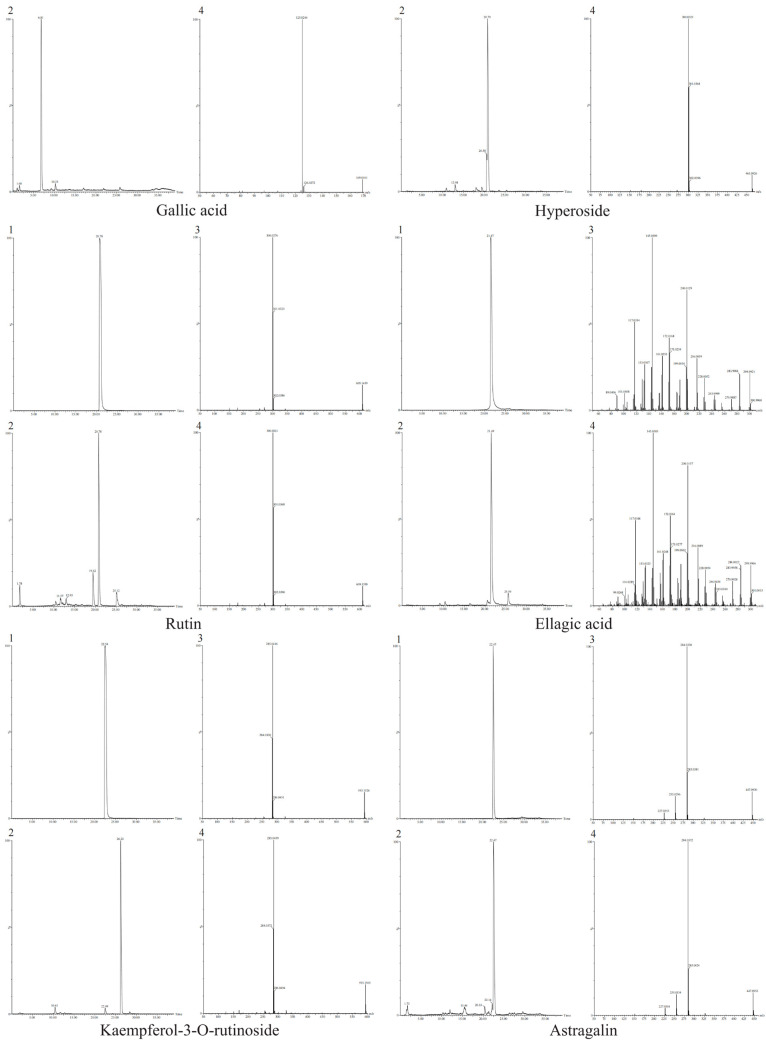

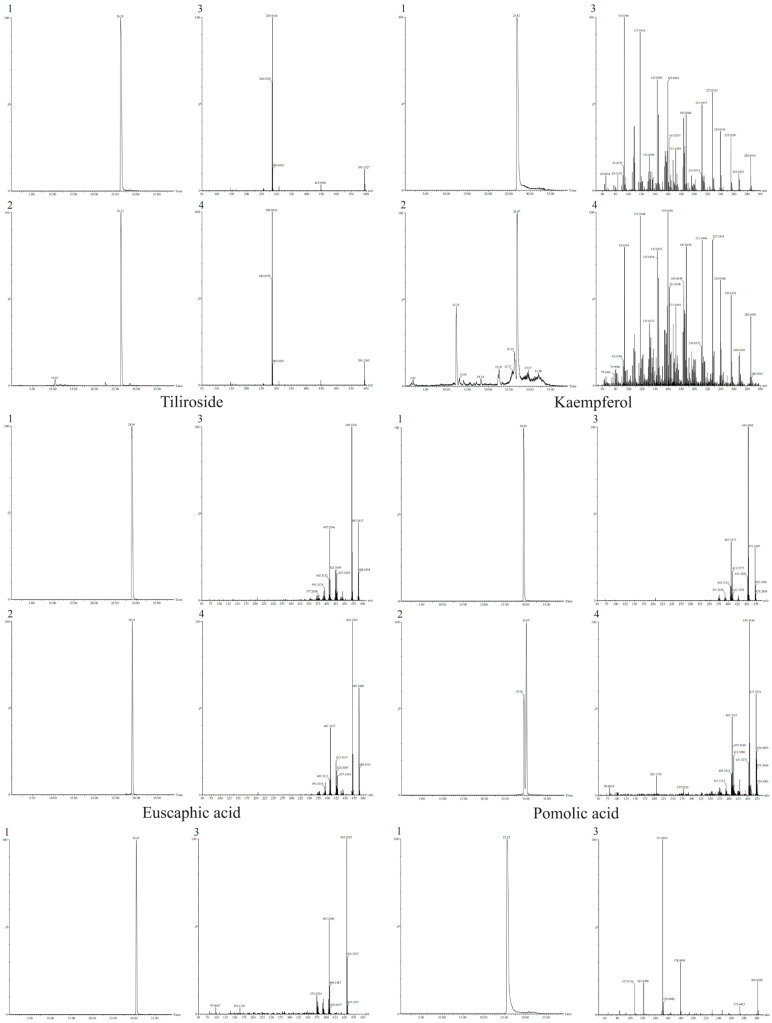

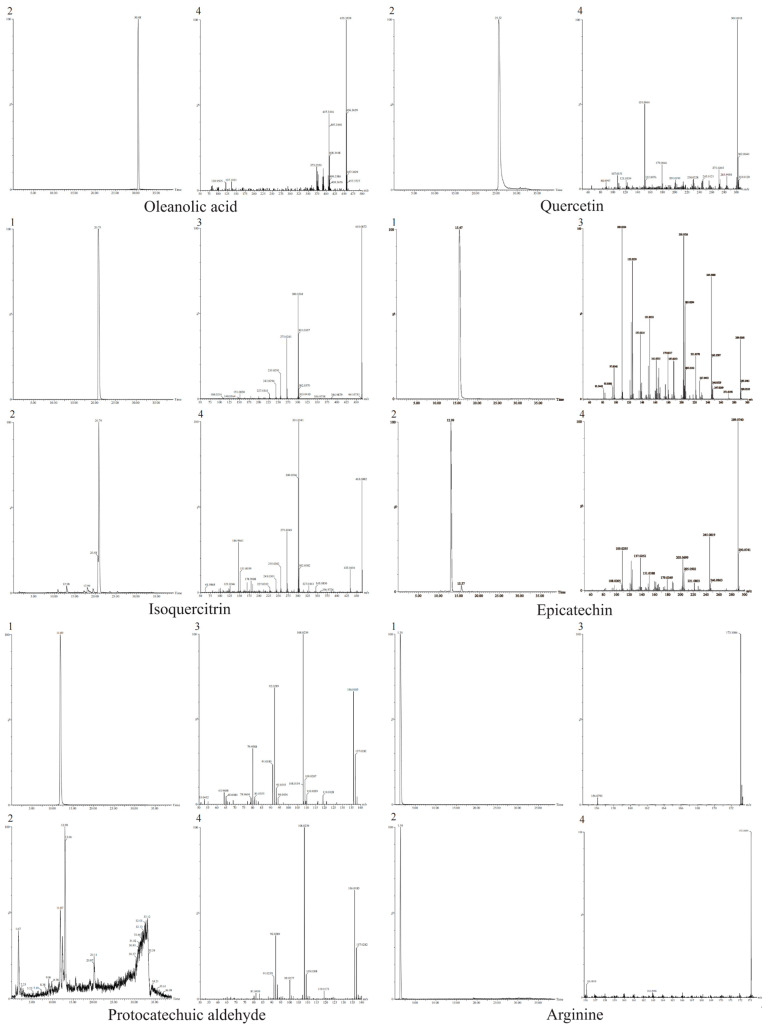
The mass spectra and chromatograms of the compounds in the reference substance and the sample. (1) The chromatogram of the reference compound. (2) The chromatogram of the compounds in the sample. (3) The mass spectrum of reference compound. (4) The mass spectrum of the compounds in the sample.

**Figure A2:**
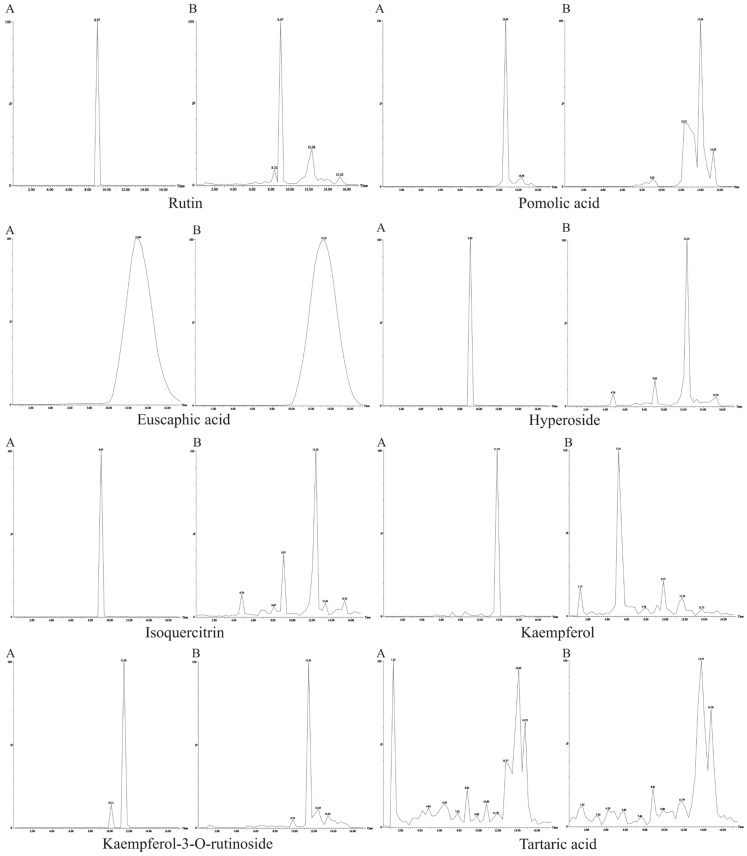

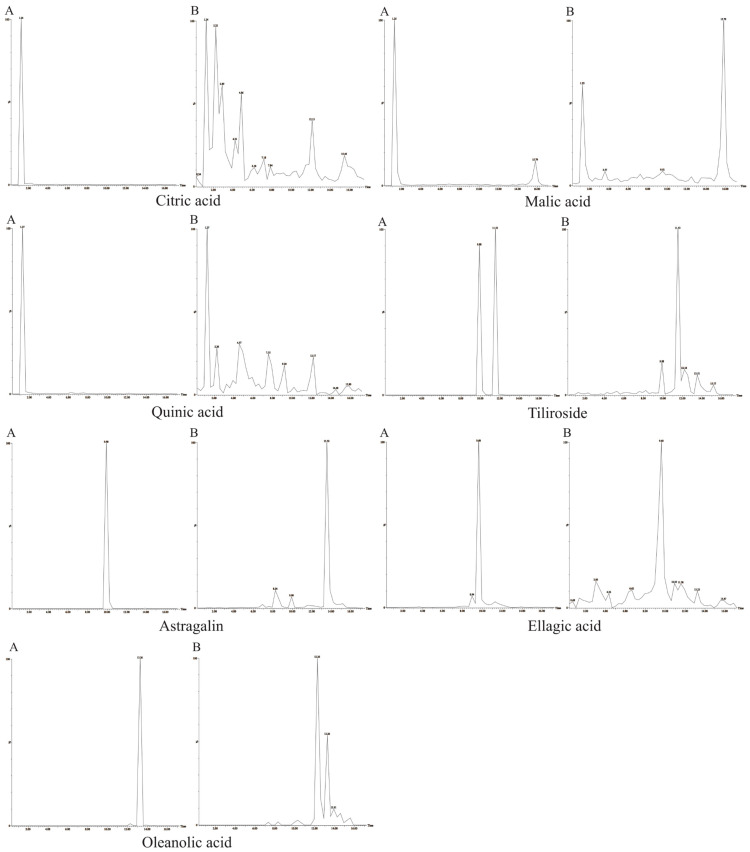
Chromatogram of blood-entering components (A) Chromatogram of blank plasma spiked with reference substances. (B) Chromatogram of compounds in the drug-containing plasma.

**Figure A3:**
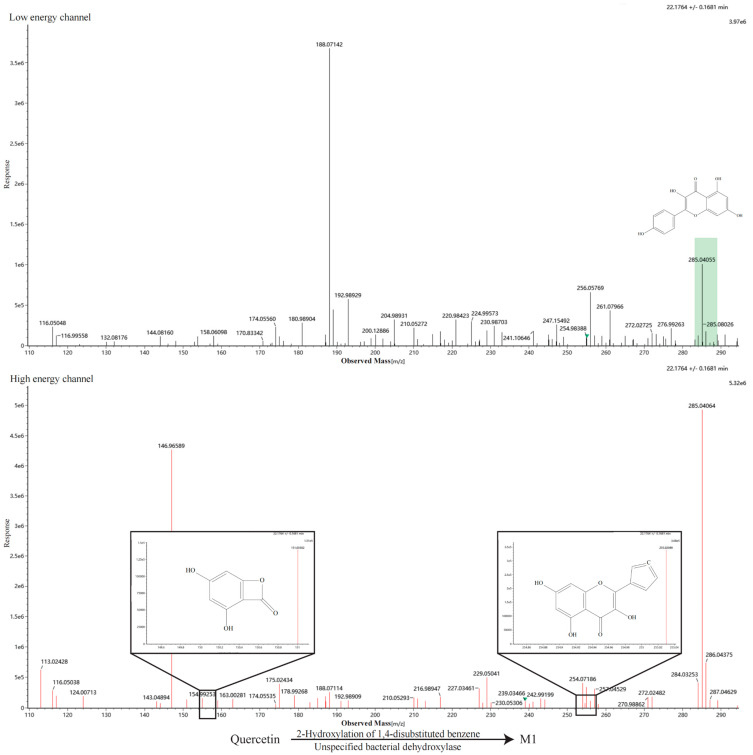

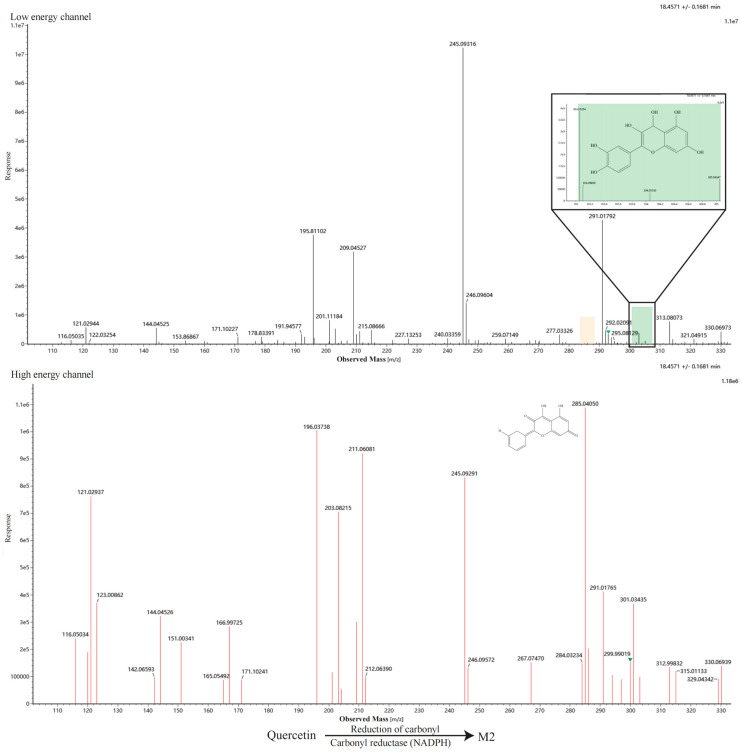

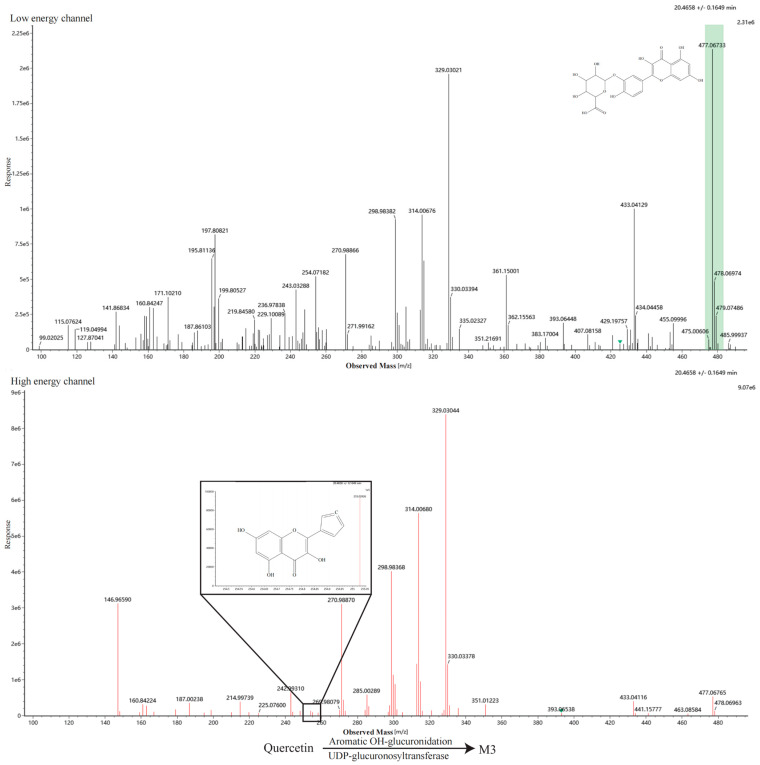

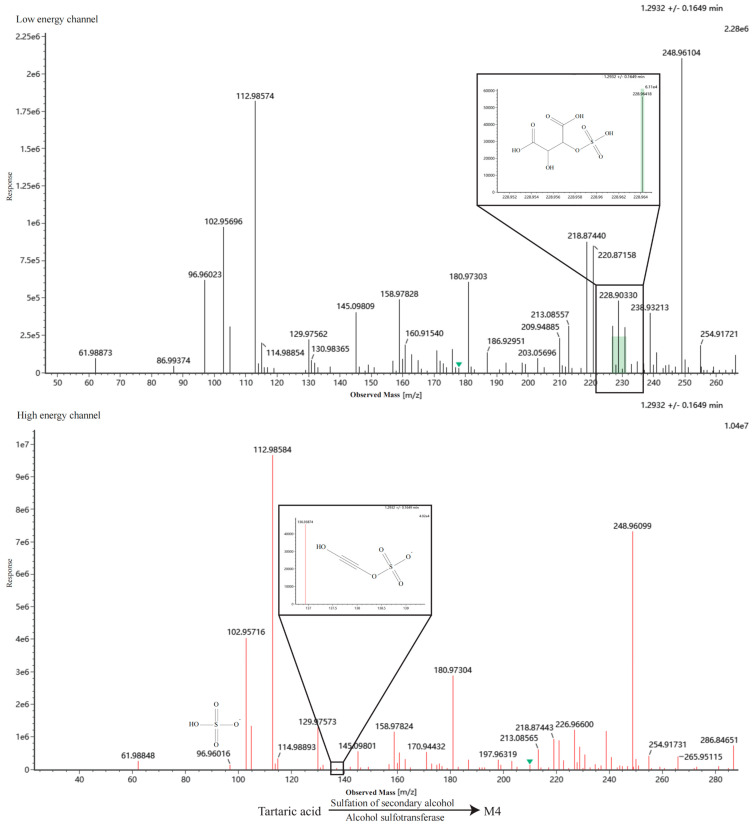

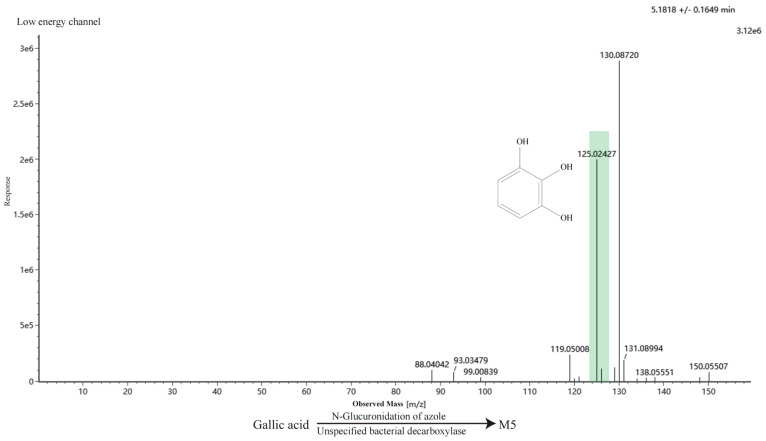

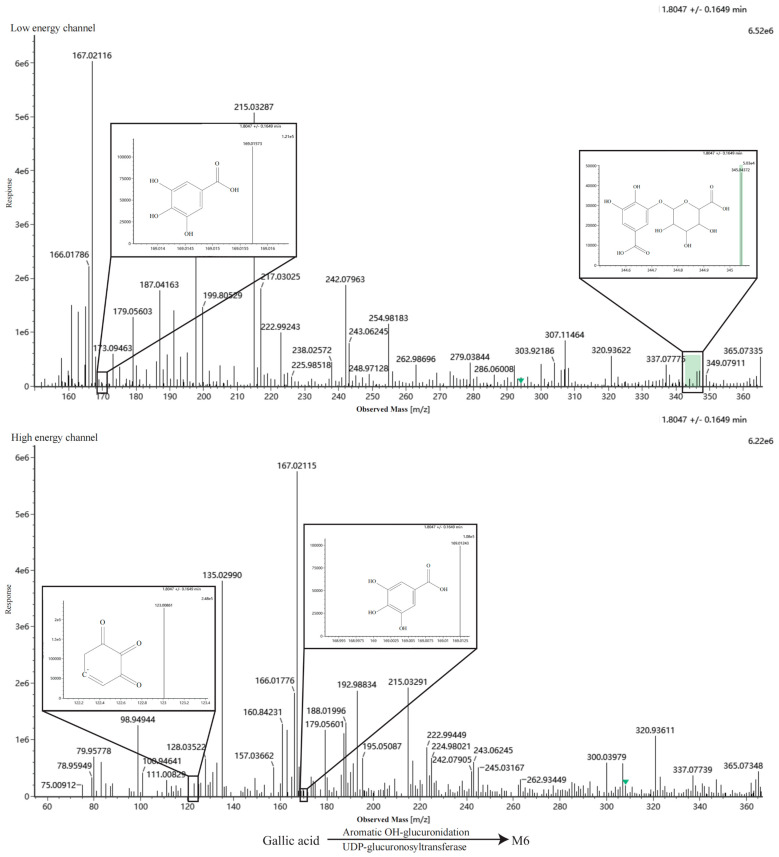

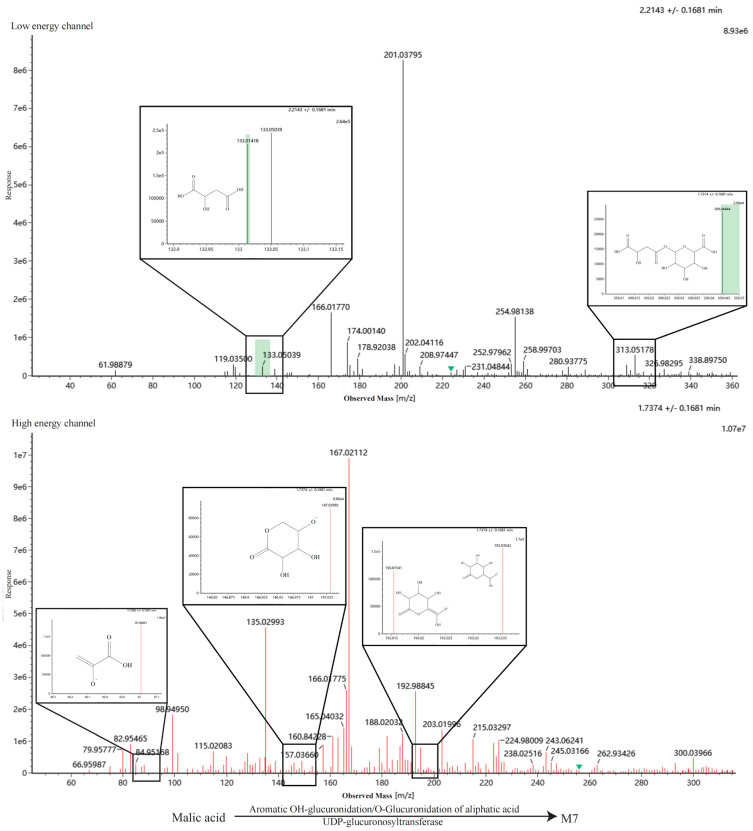

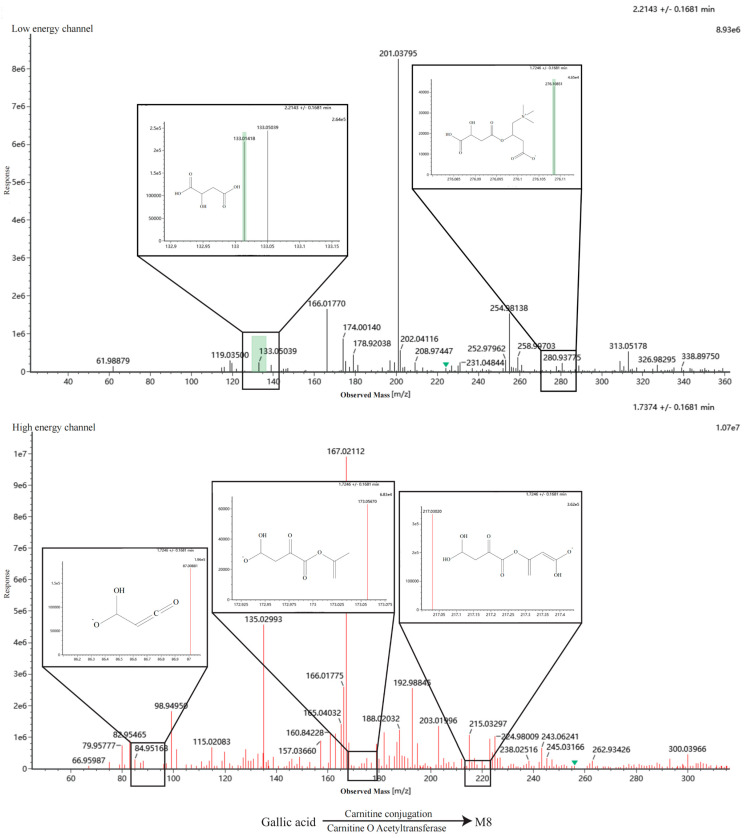

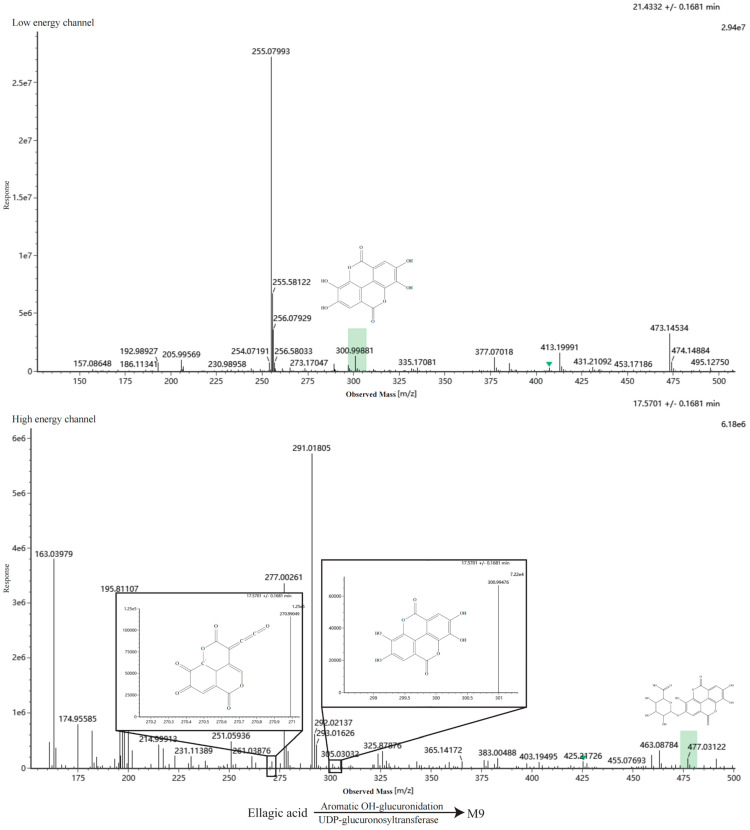

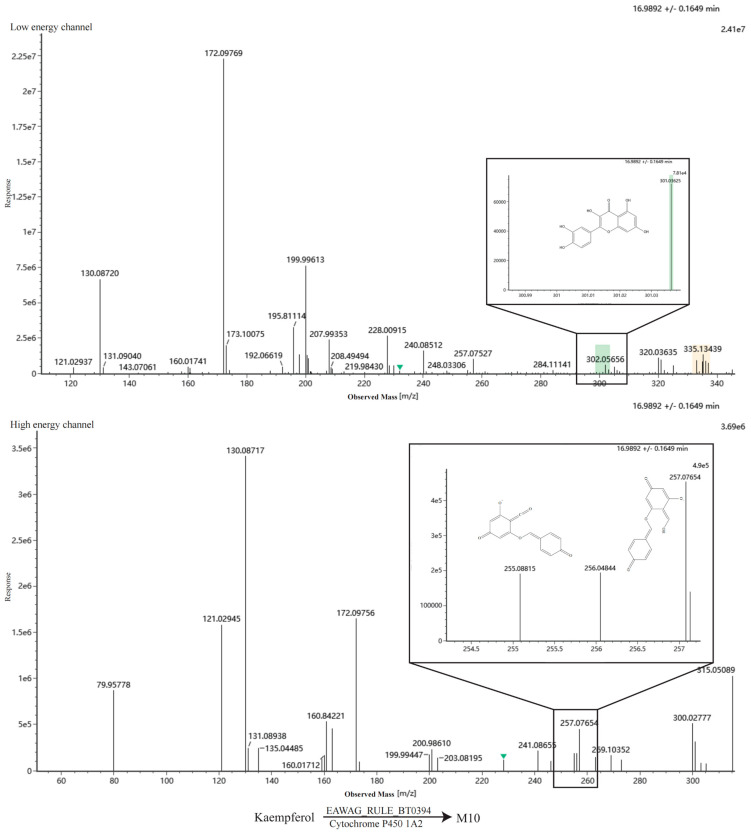

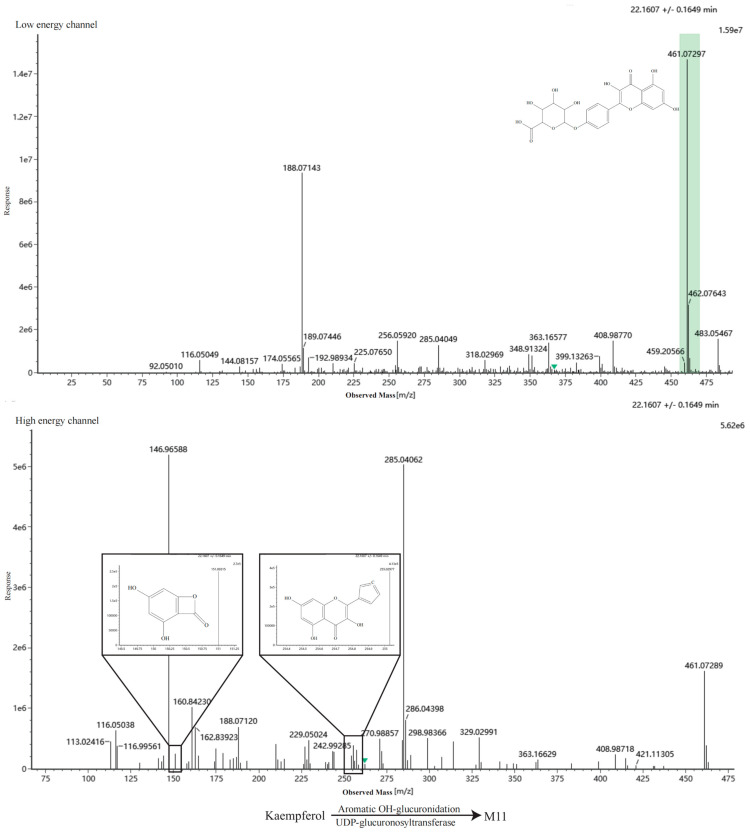

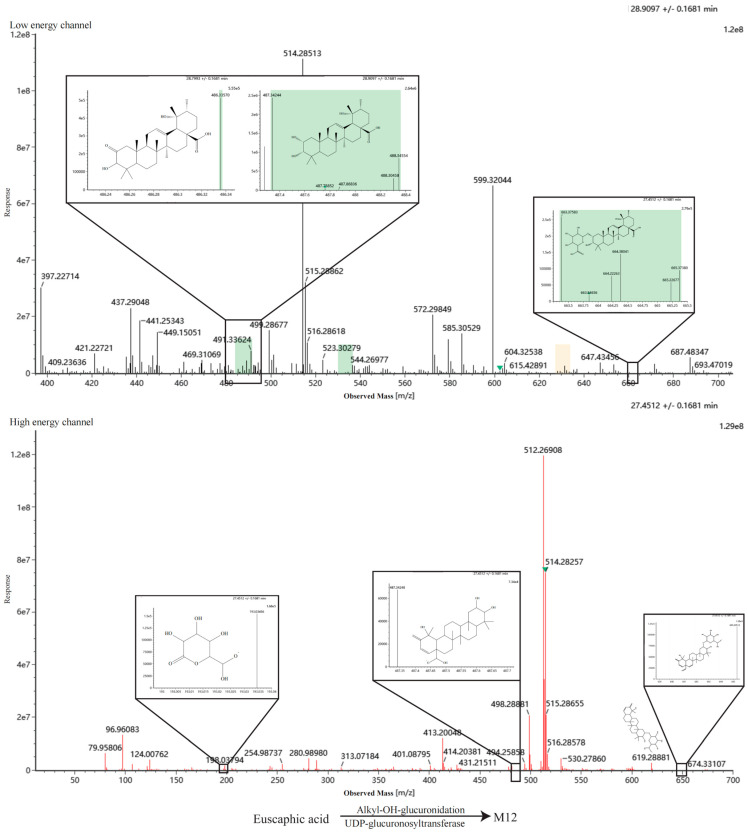

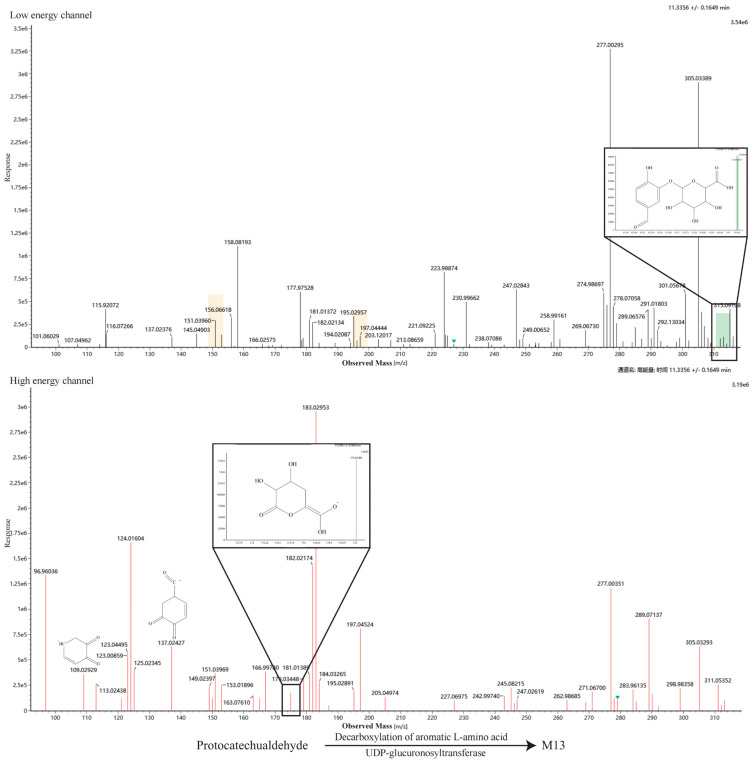

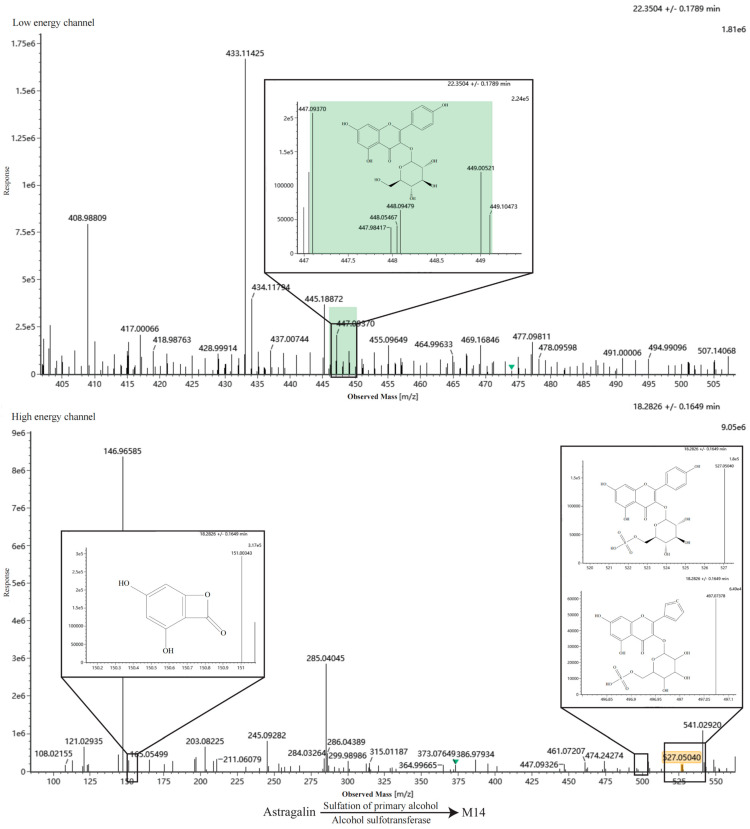
Compound in vivo metabolic product mass spectrum.

## Data Availability

The original contributions presented in this study are included in the article. Further inquiries can be directed to the corresponding author.
